# Prevalence and incidence of stroke, white matter hyperintensities, and silent brain infarcts in patients with chronic heart failure: A systematic review, meta-analysis, and meta-regression

**DOI:** 10.3389/fcvm.2022.967197

**Published:** 2022-09-15

**Authors:** Sean Tan, Clare Elisabeth Si Min Ho, Yao Neng Teo, Yao Hao Teo, Mark Yan-Yee Chan, Chi-Hang Lee, Lauren Kay Mance Evangelista, Weiqin Lin, Yao-Feng Chong, Tiong-Cheng Yeo, Vijay Kumar Sharma, Raymond C. C. Wong, Benjamin Y. Q. Tan, Leonard L. L. Yeo, Ping Chai, Ching-Hui Sia

**Affiliations:** ^1^Department of Medicine, Yong Loo Lin School of Medicine, National University of Singapore, Singapore, Singapore; ^2^Department of Cardiology, National University Heart Centre Singapore, Singapore, Singapore; ^3^Division of Neurology, University Medicine Cluster, National University Health System, Singapore, Singapore

**Keywords:** heart failure, ischemic stroke, white matter hyperintensities, silent stroke, prevalence

## Abstract

**Introduction:**

Heart failure (HF) is associated with ischemic stroke (IS). However, there are limited studies on the prevalence of IS, white matter hyperintensities (WMHs), and silent brain infarcts (SBIs). Furthermore, interaction with ejection fraction (EF) is unclear.

**Methods:**

We searched three databases (*viz*., PubMed, Embase, and Cochrane) for studies reporting the incidence or prevalence of IS, WMHs, and SBIs in HF. A total of two authors independently selected included studies. We used random-effects models, and heterogeneity was evaluated with I^2^ statistic. Meta-regression was used for subgroup analysis.

**Results:**

In total, 41 articles involving 870,002 patients were retrieved from 15,267 records. Among patients with HF, the pooled proportion of IS was 4.06% (95% CI: 2.94–5.59), and that of WMHs and SBIs was higher at 15.67% (95% CI: 4.11–44.63) and 23.45% (95% CI: 14.53–35.58), respectively. Subgroup analysis of HFpEF and HFrEF revealed a pooled prevalence of 2.97% (95% CI: 2.01–4.39) and 3.69% (95% CI: 2.34–5.77), respectively. Subgroup analysis of WMH Fazekas scores 1, 2, and 3 revealed a decreasing trend from 60.57 % (95% CI: 35.13–81.33) to 11.57% (95% CI: 10.40–12.85) to 3.07% (95% CI: 0.95–9.47). The relative risk and hazard ratio of patients with HF developing IS were 2.29 (95% CI: 1.43–3.68) and 1.63 (95% CI: 1.22–2.18), respectively. Meta-regression showed IS prevalence was positively correlated with decreasing anticoagulant usage.

**Conclusion:**

We obtained estimates for the prevalence of IS, WMH, and SBI in HF from systematic review of the literature.

**Systematic review registration:**

https://www.crd.york.ac.uk/prospero/display_record.php?RecordID=255126, PROSPERO [CRD42021255126].

## Introduction

HF is a global health problem, and its associated socioeconomic burden is enormous (1). It is widely assumed that HF predisposes individuals to the development of thromboembolic complications (2). In Virchow's triad, reduced cardiac output contributes to venous stasis, and the ensuing compensatory neuroendocrine mechanisms culminate in a hypercoagulable state (3, 4). Last, abnormal levels of von Willebrand factor (VWF) (3, 4) and endothelium-derived nitric oxide lead to endothelial dysfunction (5). These result in complications such as ischemic stroke (IS) (6) and stroke precursors including white matter hyperintensities (WMHs) and silent brain infarcts (SBIs) (7, 8).

To date, the magnitude of the problem of HF has not been comprehensively assessed due to marked variability in population-based estimates of the prevalence and incidence of associated complications (9). In recent years, few meta-analyses have explored the link between HF and its complications of IS (10–12), SBIs, and WMHs, with most stating similar limitations. A recent study by Witt et al. cited the lack of data on left ventricular ejection fraction (LVEF) as a major limitation of their meta-analysis (12). One of the independent risk factors associated with IS is impairment of left ventricular systolic function (13). Apart from IS, HF also predisposes individuals to an increased risk of other cerebrovascular outcomes, such as WMH and SBI. Currently, WMH has been gaining increased attention due to its prognostic role as a strong risk factor for stroke (14). However, not much has been described on the relationship between HF and WMH as these were previously considered an incidental finding on cerebral magnetic resonance imaging (MRI) with little therapeutic consequence (15). Our study sought to analyze the prevalence of IS in both patients with HF and subgroups of patients with different levels of ejection fraction. We also sought to quantify the prevalence of WMH in patients with HF as they both share similar pathophysiological mechanisms, including small-vessel disease (SVD) and large-vessel atherosclerosis (16).

Similarly, SBIs, defined as infarctions in the brain parenchyma without a history of stroke and only detected by MRI, have also been associated with HF (7, 8). The wide prevalence of SBI has been estimated to far exceed symptomatic stroke, with 10 cases of SBI for every one symptomatic stroke (17) and ~25% of individuals older than 80 years have SBI (18). The resultant higher risk of strokes was mostly ischemic in nature at 89% (19). Previously, Siachos et al. demonstrated a 34% prevalence of SBI in a trial of heart failure with reduced ejection fraction (HFrEF) patients only (20). Hence, while the exact relationship between HF and SBI remains unclear, our study seeks to quantify this important relationship with the aim of intervening before SBI develops into clinically overt IS.

Many studies explored the potential prophylactic role of anticoagulants in the development of IS among patients with HF but arrived at differing conclusions. The most recent meta-analysis by Redding et al. found an association between anticoagulant usage and the prevalence of IS through analysis of pooled odds ratio of four studies and 9,000 patients. Specifically, with regard to the use of newer oral anticoagulant (NOAC), the recent COMMANDER-HF trial suggested promising reduction of stroke events with low-dose rivaroxaban in a unique population of patients who suffered a recent episode of worsening HFrEF. However, the use of anticoagulants in patients with HF is a topic that should not be taken lightly, especially with its associated bleeding risks. Meta-regression is an analytical tool to derive a regression model to quantify the correlation between factors and to account for heterogeneity between studies. Our meta-analysis seeks to shed light on the highly debated role of anticoagulant use in patients with HF through a rigorous means of analysis with the use of meta-regression of proportions, which, to the best of our knowledge, has not been used in the existing literature.

Therefore, our study aims to address the aforementioned knowledge gaps by comprehensively summarizing and analyzing existing data on the development of WMH, SBI, and IS in patients with HF. Furthermore, quantifying the prevalence of WMH and SBI as asymptomatic precursors of IS may highlight the significance of early screening and intervention.

## Materials and methods

### Study identification

Ethics approval and consent to participate were not required as this was a systematic review of previously published data. The data that support the findings of this study are publicly available in the published literature. The meta-analysis was registered on PROSPERO (CRD42021255126) and reported according to the 2020 Preferred Reporting Items of Systematic Reviews and Meta-Analyses (PRISMA) guidelines (21). Studies were identified by searching across three electronic databases, namely, PubMed, Embase, and Cochrane, for articles published from inception to 2 May 2021. The search terms can be found in Appendix A in Supplementary material. The inclusion and exclusion criteria of our results are listed in Table 1, and only English language articles were included. In addition, studies were identified by hand-searching the reference list of relevant studies.

**Table 1:** PECOS, inclusion criteria, and exclusion criteria for database search.

**PECOS**	**Inclusion criteria**	**Exclusion criteria**
Population	• Patients with chronic HF and IS and/or WMH and/or SBI	• Studies with the study population suffering from the co-morbidity of stroke independent of HF
		• Studies which did not distinguish between IS and hemorrhagic stroke
		• Studies with outcomes did not distinguish between systemic embolism and IS
Exposure	• Cerebrovascular ischemic events occurring 15 years after the diagnosis of HF	
Comparison	• HFpEF	• Hemorrhagic Stroke
	• HFrEF	
	• IS	
	• WMH	
	• SBI	
Outcome	• IS and its subtypes (Thrombosis, embolism, hypoperfusion)	
	• SBI as hypointense lesions 3mm or larger on T1-weighted images (WI) and hyperintense on T2-weighted images on brain MRI	
	• Fazekas scale for WMH on T2-weighted MRI (22)	
Study outline	• Articles in English or translated to English	• Case reports, case series and non-original research articles
	• Randomized clinical trials	• Mixed methods research, meta-analysis, systematic reviews and descriptive papers
	• Cohort studies	
	• Case-control studies	
	• Cross-sectional studies	
	• Year of publication: Inception– 2 May, 2021	
	• Databases: PubMed, Embase, Cochrane	

A total of two reviewers determined study eligibility and independently used the planned search strategy to search for relevant literature and data extraction. Discussion and consensus with a third reviewer resolved all disagreements. Studies were selected based on the inclusion criteria during the title and abstract reviews. Duplicate studies and studies explicitly meeting the exclusion criteria such as non-original research articles were excluded. Randomized clinical trials and cohort studies involving patients with chronic HF were chosen for the full-text review stage to determine if the studies reported the cerebrovascular outcomes of patients with HF, if they distinguished between ischemic and hemorrhagic strokes, and if the study populations were not suffering from the comorbidity of stroke independent of HF.

### Data extraction

Data extracted are shown in Table 2. We collected data on cerebrovascular outcomes and baseline characteristics of participants including age and gender; comorbidities such as atrial fibrillation, diabetes mellitus, hypertension, hyperlipidemia, past myocardial infarction, and past stroke; and medications, such as antiplatelets, anticoagulants, and statins. The data collection sheet also recorded the total study cohort size, HF definition used, and the duration of follow-up. For the results on the prevalence of IS, SBI, and WMH, we collected data on the number of IS, WMH, and SBI events experienced by patients with HF. The number of IS events in heart failure with preserved ejection fraction (HFpEF) and HFrEF patient groups was also collected if reported. For results on the incidence of IS, hazard ratios and risk ratios for IS in patients with HF were collected in studies which reported them.

**Table 2:** Baseline characteristics of the included studies.

**References**	**Total patients**	**Gender distribution (M)**	**Age/year**	**Concurrent AF**	**Previous stroke**	**Anti-platelet**	**Anti-coagulation**
**Included studies with IS outcomes**							
Adelborg et al. (23)	289,353	51.96%	77	10.70%	NR	NR	NR
Alberts et al. (24)	1,247	NR	NR	NR	NR	NR	NR
Berger et al. (25)	66,414	50.00%	68	21.70%	17.90%	NR	NR
Berkovitch et al. (26)	2,922	50.00%	79	100.00%	NR	NR	89.84%
Chi et al. (27)	7,513	44.86%	76	36.43%	10.40%	57.03%	100%
Chou et al. (28)	12,179	45.40%	66	0%	NR	NR	1.03%
de Peuter et al. (29)	20,870	49%	75	NR	NR	NR	NR
Friberg etl al. (30)	92,532	54.20%	79	100.00%	16.40%	52.00%	45.30%
Greenberg et al. (31)	7,005	52.00%	73	22.30%	7.60%	10.60%	25.50%
Hamatani et al. (32)	721	60.00%	76	54.00%	25.00%	36.00%	33.00%
Hjalmarsson et al. (33)	15,425	61.30%	73	NR	9.00%	72.80%	26.10%
Iguchi et al. (34)	338	51.50%	78	100.00%	22.80%	NR	68.00%
Kang et al. (35)	5,746	41.00%	70	NR	16.30%	51.10%	10.20%
Kim et al. (36)	1,869	NR	43	100.00%	NR	NR	NR
Komori et al. (37)	111	78.20%	67	31%	NR	NR	40%
Kondo et al. (38)	127	76.40%	64	NR	NR	33.85%	42.80%
Lip et al. (39)	1,309	37.80%	67	0.00%	NR	8.94%	69.00%
Loh et al. (40)	2,231	82.52%	59	NR	NR	58.72%	28.33%
McMurray et al. (41)	5,943	67.50%	68	100.00%	16.69%	35.15%	100.00%
Mehra et al. (42)	5,022	57.37%	66	0%	9.02%	93%	97%
Melgaard et al. (43)	42,987	55.30%	74	21.90%	8.75%	48.64%	0.00%
Merkler et al. (44)	7,848	65.58%	53	30.45%	NR	NR	NR
Nakano et al. (45)	92,573	48.20%	81	NR	NR	38.70%	23.71%
Nakayama et al. (46)	191	46.07%	75	41.88%	NR	NR	NR
Qualls et al. (47)	8,558	50.84%	78	57.20%	20.69%	26.91%	100%
Shintani et al. (48)	950	61.00%	77	51.00%	NR	45.00%	67.00%
Tai et al. (49)	18,373	50.10%	75	8.60%	69.60%	NR	27.50%
Tseng et al. (50)	287	84%	68	80.10%	NR	NR	100%
Tütüncü et al. (51)	2,248	73.44%	71	31.45%	NR	39.90%	56.80%
Vemmos et al. (52)	2,904	67.10%	70	50.90%	NR	49.00%	36.00%
Witt et al. (53)	630	46.10%	76	41.42%	11.74%	34.76%	18.10%
Wolsk et al. (54)	136,545	53.00%	73	NR	NR	NR	NR
Yusuf et al. (55)	3,023	59.86%	67	29.14%	8.87%	59.84%	23.34%
Zhirov et al. (56)	1,003	56.40%	68	100.00%	15.80%	46.50%	68.60%
Zhou et al. (57)	9,485	70.90%	66	21.10%	4.30%	41.40%	11.50%
**Included studies with outcomes of SBI**							
Davis et al. (58)	618	61.30%	66	NR	NR	NR	NR
Kozdag et al. (13)	72	73.61%	62	NR	NR	77.78%	NR
Oliveira et al. (59)	75	56.00%	61	17.30%	NR	54.70%	25.30%
Siachos et al. (20)	117	74.00%	51	NR	NR	26.00%	27.00%
**Included studies with outcomes of WMH**							
Frey et al. (60)	148	84.50%	65	21.60%	NR	57.40%	30.40%
Stegmann et al. (61)	2,490	54.00%	64	NR	NR	NR	NR

### Statistical analysis

Pooled mean prevalence with 95% confidence intervals was calculated for IS, SBI, and WMH. Analyses were performed in RStudio (version 1.4.1106), unless stated otherwise. Meta-analysis of the proportions was performed with a one-step approach using a generalized linear mixed model (GLMM) method with a logit transformation using the function “metaprop.” In comparison to traditional two-stage methods, the one-step GLMM approach is proven to report less biased estimates, smaller errors, and greater coverage probabilities (62, 63). No continuity correction was applied, and all analyses were conducted using a random-effects model to account for methodological heterogeneity present between studies. The I^2^ statistic was used as an assessment of between-study variation, with an increasing I^2^ value representing greater levels of heterogeneity, and values of <30, 30–60, and >60% indicating low, moderate, and substantial levels of heterogeneity, respectively (64). For incidence rates, the pooled risk ratio (RR) and hazard ratio (HR) were quantitatively pooled and analyzed using Review Manager (RevMan) version 5.4, using general approaches mentioned in the Cochrane Handbook (65). To explore sources of heterogeneity, we conducted subgroup analysis. Where a minimum of two studies per subgroup was available, subgroup analyses were performed for the following studies with IS outcomes: HFrEF and HFpEF, less than and more than 1-year follow-up. For studies with WMH outcomes, subgroup analysis was performed according to the Fazekas scale (1, 2, and 3), which grades periventricular white matter and deep white matter lesions depending on the size and confluence of the lesions (22). For studies with SBI outcomes, less than and more than 1-year follow-up subgroups were performed. For subgroups that had two points of comparison, the Z-test was used to compare pooled prevalence data. For subgroups with more than two points of comparison, a Q-test was performed based on analyses of variance. A *p* < 0.05 was considered significant.

To account for heterogeneity between studies, meta-regression analysis was performed on variables including medication and comorbidities of patients. Antiplatelets have been widely used as prophylaxis in patients with coronary artery disease to reduce the risk of myocardial infarction and IS (66), while anticoagulation is used to lower the risk of cardioembolic stroke in patients with atrial fibrillation (67). In addition, comorbidities that have been previously known to have a strong correlation to the development of ischemic stroke was adjusted for including hypertension (68), diabetes mellitus (69), hyperlipidemia (70), and atrial fibrillation (71). Patients with prior strokes (72) or myocardial infarction (73) have also shown to be at an increased risk of ischemic stroke. The function “metareg” was used to perform a mixed-effect model meta-regression for years following HF diagnosis and anticoagulant usage. The size of the bubbles is proportional to the weights assigned to the studies. The upper and lower confidence intervals are also drawn within the figures in dotted lines. A gradient with a *p* < 0.05 was considered to represent significant correlation between the variables. To account for heterogeneity between studies, meta-regression analysis was performed on variables including medication and comorbidities of patients. Antiplatelets have been widely used as prophylaxis in patients with coronary artery disease to reduce the risk of myocardial infarction and IS, while anticoagulants are used to lower the risk of cardioembolic stroke in patients with atrial fibrillation. The function “metareg” was used to perform a mixed-effect model meta-regression for years following HF diagnosis and anticoagulant usage. The size of the bubbles is proportional to the weights assigned to the studies. The upper and lower confidence intervals are also drawn within the figures in dotted lines. A gradient with a *p* < 0.05 was considered to represent significant correlation between the variables.

Publication bias of studies was assessed using funnel plots, where the presence of asymmetry in the distribution of studies suggests bias (74). Quantitative assessment of funnel plot asymmetry was performed with linear regression analysis utilizing Egger's regression test, based on unweighted linear regression (75). An unweighted regression test was chosen over the traditional weighted test as it is no longer advocated due to the lack of theoretical justification (76). Egger's regression test was selected over a ranked correlation test as the latter is only powerful for large meta-analyses involving more than 75 studies (76).

### Quality of evidence

The Newcastle–Ottawa Scale for cohort studies was used to assess the quality and risk of bias in included studies, which is summarized in Table S1 in Supplementary material. The scale assesses three main categories of selection (representativeness of the exposed cohort, selection of the non-exposed cohort, ascertainment of exposure, demonstration that the outcome of interest was not present at the start of the study), comparability (comparability of cohorts on basis of design and analysis), and exposure (assessment of outcome, if the length of the follow-up was long enough for outcomes to occur, adequacy of follow-up of cohorts). Studies could earn a maximum of nine star-points, with a maximum of 1-star point for each item within selection and outcome categories and a maximum of 2-star point given for the comparability category (77, 78). Under the adequacy of follow-up items in the outcome category, we determined a loss to follow-up of <5% as being unlikely to introduce bias. On the other hand, we suspected bias in studies with a loss of follow-up of more than 5% or an unreported loss to follow-up data.

## Results

**Figure 1:**
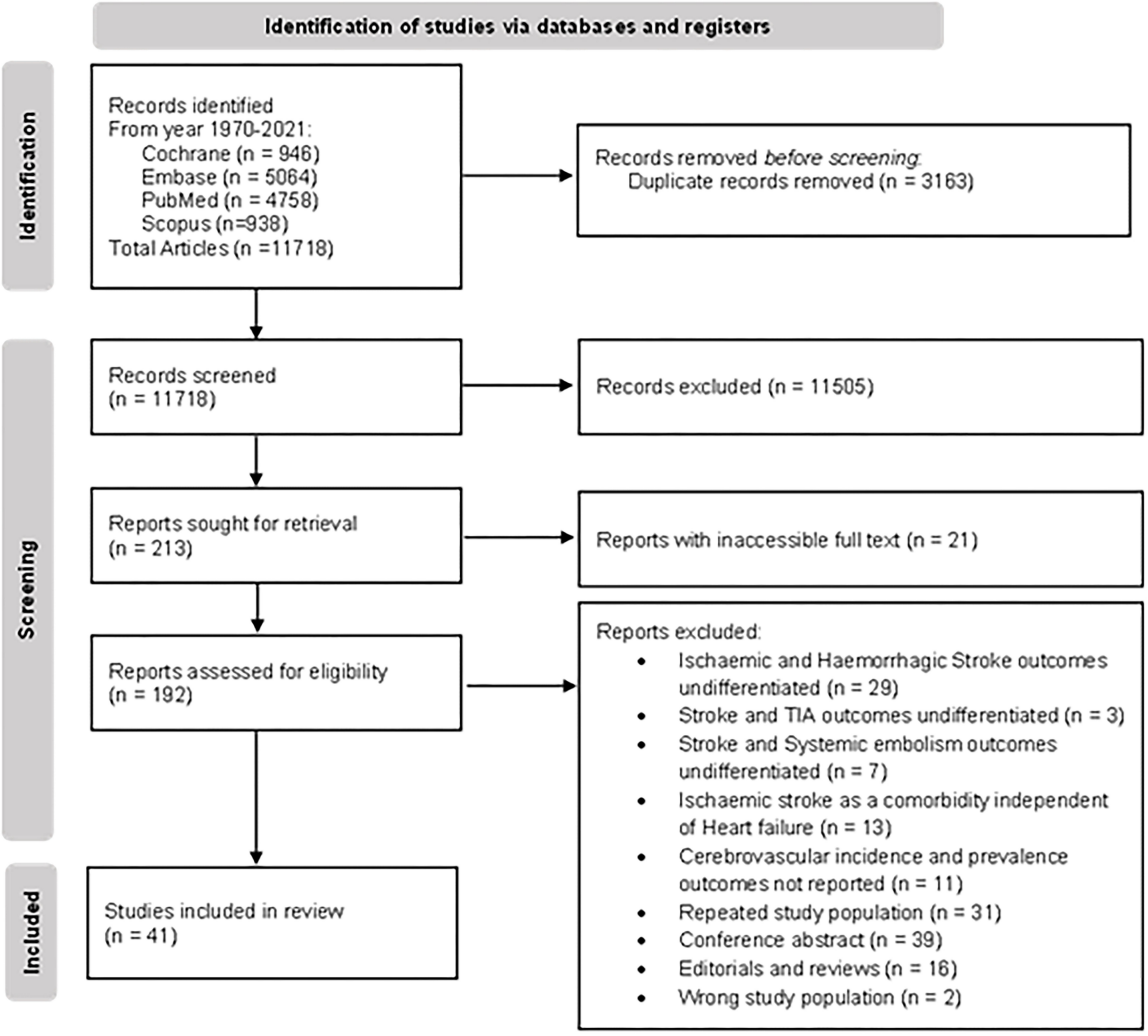
PRISMA flowchart of study selection.

The literature search of PubMed, Embase, and Cochrane retrieved 15,267 results. After removing duplicate studies, the title and abstracts of the remaining 11,718 articles were screened; 11,505 records were excluded during the screen for reasons, namely, the study population did not include patients with HF, results did not report any cerebrovascular outcomes, or the studies were of an inappropriate type. The full-text screening excluded a further 151 articles. A total of 41 articles were included for the review and meta-analysis (20, 23–61, 79). The Preferred Reporting Items for Systemic Reviews and Meta-Analyses (PRSIMA) flowchart is presented in Figure 1.

### Baseline characteristics

The baseline characteristics of the included studies comprising a total of 870,002 patients are summarized in Table 2. Across the 41 studies, comorbidities including atrial fibrillation (AF), previous stroke, and concurrent treatment including antiplatelet and anticoagulation therapy are also summarized in Table 2. Other comorbidities and treatments are available in the Table S2 in Supplementary material.

#### IS prevalence in patients with HF

**Figure 2:**
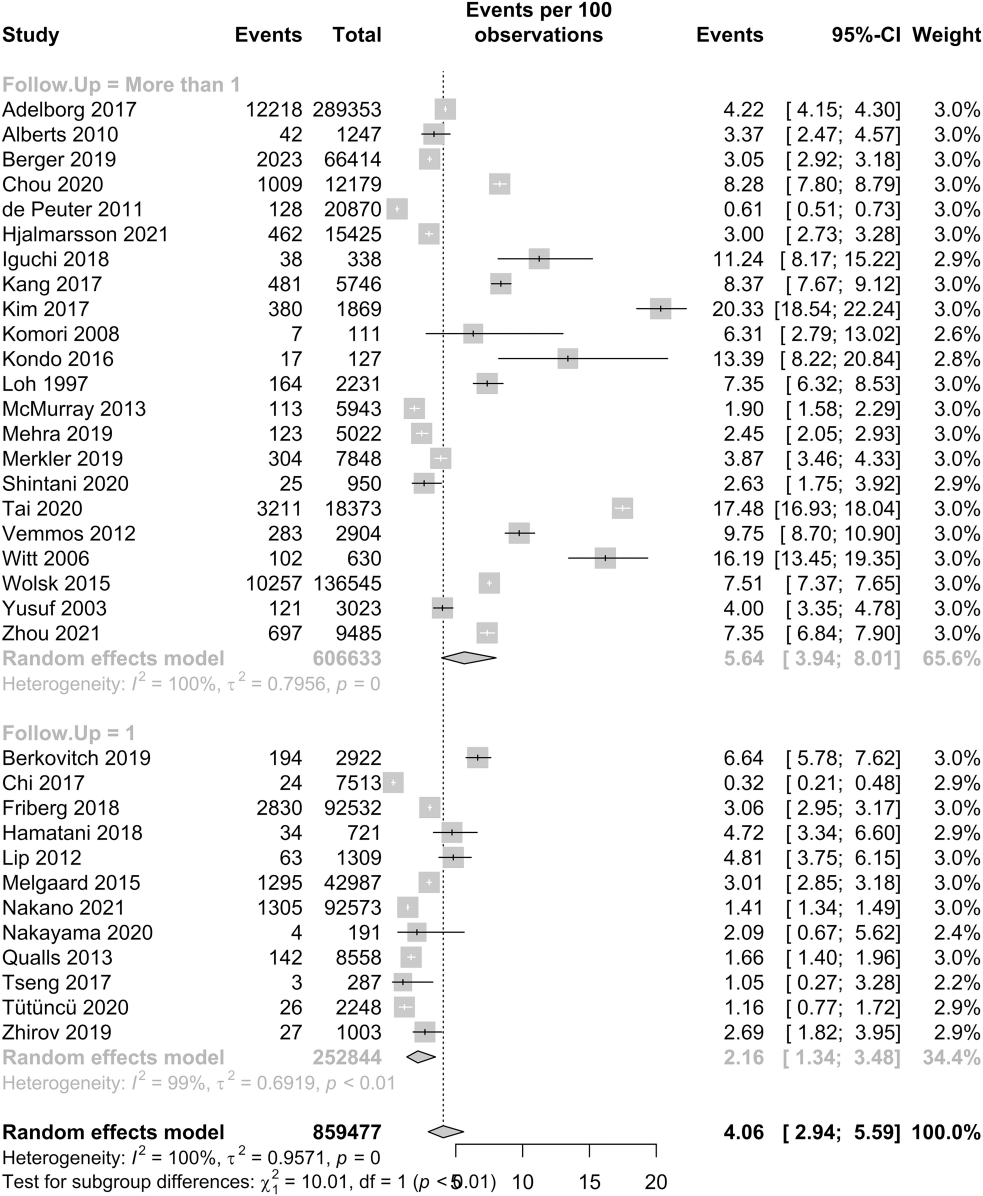
Forest plot of the prevalence of IS in HF patients.

Our meta-analysis included 35 studies, with a total of 866,482 patients, and the prevalence of IS in patients with HF is reported in Figure 2. In patients with HF, the pooled proportion of IS was 4.06% (95% CI: 2.94–5.59, I^2^ = 100%). In our subgroup analysis, there was a significant difference in the pooled proportion of IS at a follow-up period of 1 year and more than 1 year, which were 2.16 and 5.64%, respectively. The *p*-value was <0.01. The *I*^2^ statistic was 100%, which indicates that the heterogeneity of our studies was high.

#### HFpEF and HFrEF and time dependency of stroke risk

**Figure 3:**
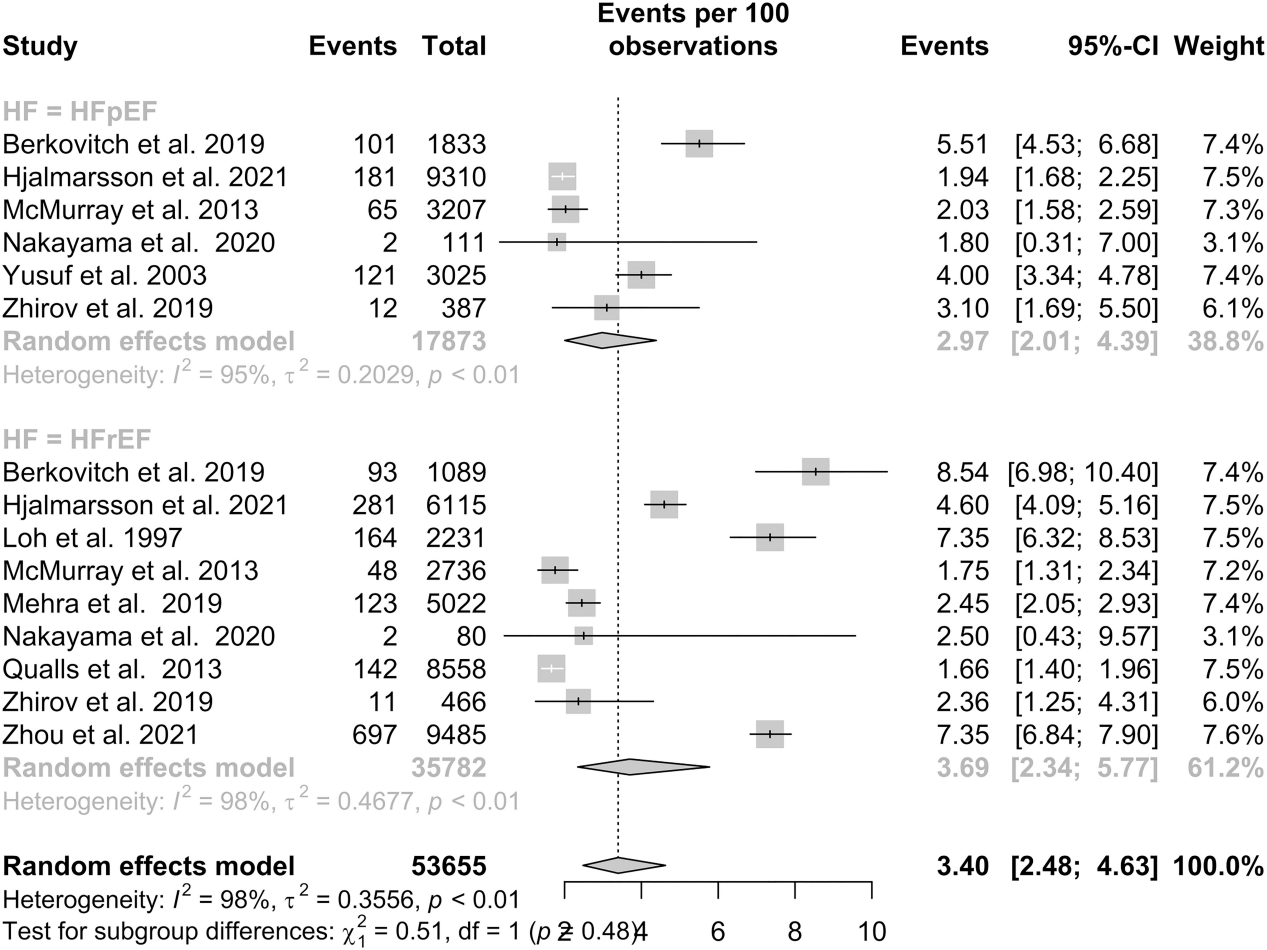
Subgroup analysis of prevalence of IS in patients with HFpEF and HFrEF.

A total of six studies reported IS outcomes in patients with HFpEF, which had a pooled proportion of 2.97% (95% CI: 2.01–4.39). Subgroup analysis in Figure 3 showed that the pooled proportions over a follow-up period of 1 year or less and more than 1 year were 3.88% (95% CI: 2.20–6.76) and 2.51% (95% CI: 1.58–3.98), respectively. A total of nine studies reported IS outcomes in patients with HFrEF, which had a pooled proportion of 3.69% (95% CI: 2.34–5.77). Subgroup analysis showed that the pooled proportions over a follow-up period of 1 year or less and more than 1 year were 3.15% (95% CI: 1.38–7.03) and 4.09% (95% CI: 2.32–7.13), respectively. Our subgroup analysis demonstrated that there was no statistically significant difference in IS between the two groups, with a *p*-value of 0.48.

#### IS incidence in patients with HF

There was an association between patients with HF and the incidence of subsequent IS. In all, three studies reported the risk ratio (RR), and 10 studies reported the hazard ratio (HR) for HF and IS. The pooled RR was 2.29 (95% CI: 1.43–3.68), and pooled HR was 1.63 (95% CI: 1.22–2.18).

#### Silent brain infarct prevalence in patients with HF

**Figure 4:**
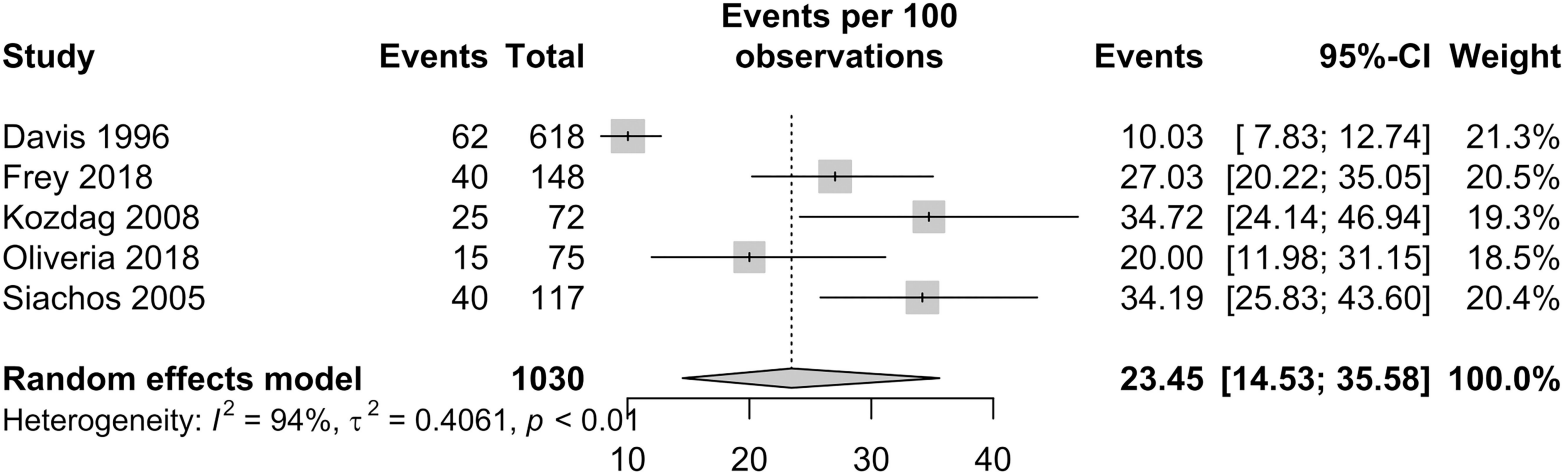
Forest plot of incidence of silent brain infarctions in patients with HF.

The prevalence of SBI in patients with HF is presented in Figure 4. In patients with HF, the pooled proportion of SBI was 23.45% (95% CI: 14.53–35.58). Within studies that had a follow-up period of more than a year, the pooled proportion of SBI was 21.13% (95% CI: 11.97–34.55). Only one study by Siachos et al. had a follow-up period of less than a year, with a proportion of SBI reported at 34.19% (20).

#### White matter hyperintensity prevalence in patients with HF

**Figure 5:**
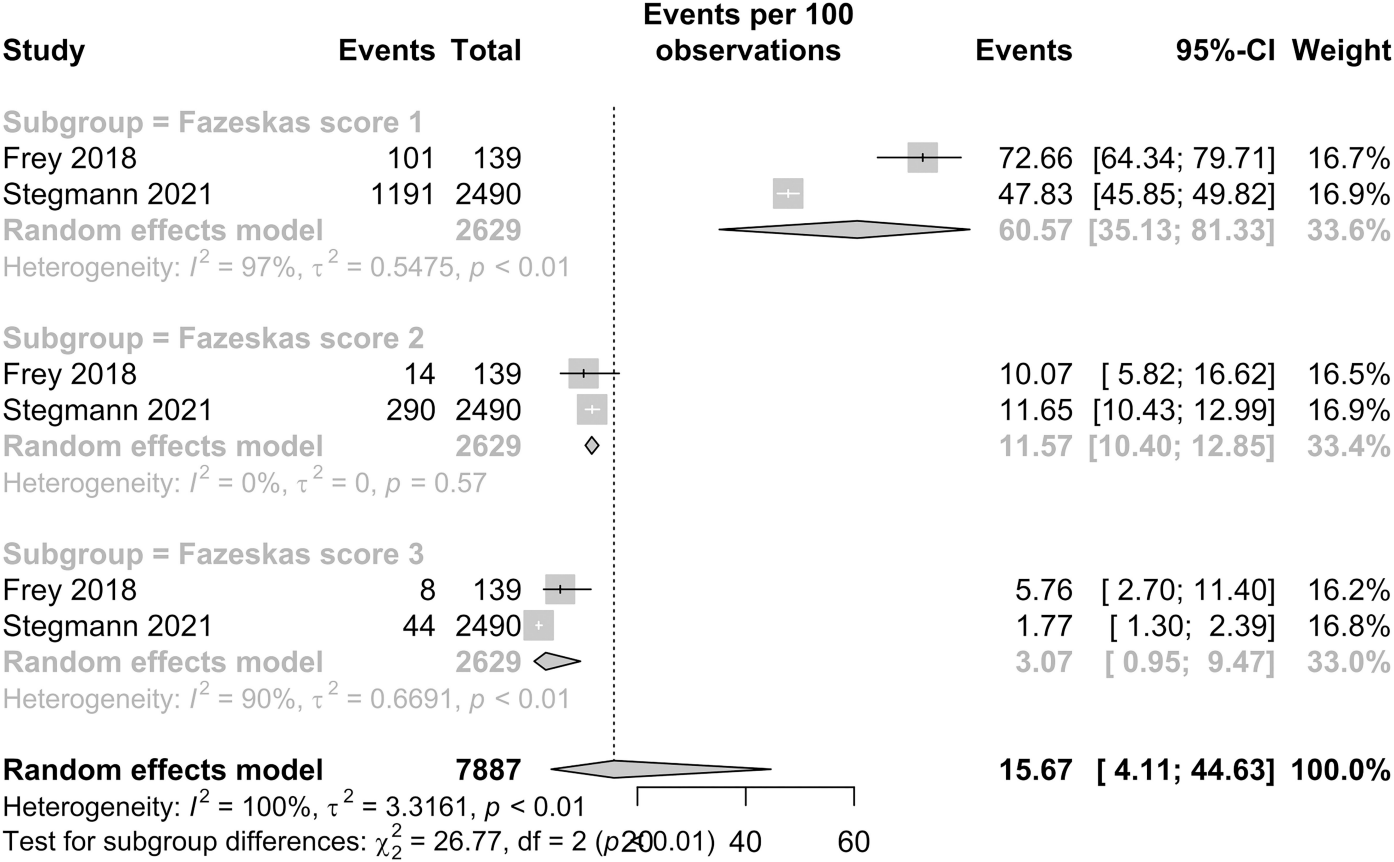
Forest plot of prevalence of WMH (Fazekas scores 1, 2, and 3) in HF patients.

The prevalence of WMH in patients with HF is presented in Figure 5. Among studies with a Fazekas score of 1, the pooled proportion of WMH was 60.57% (95% CI: 35.13–81.33). Among studies with Fazekas scores of 2 and 3, the pooled proportion of WMH was 11.57% (95% CI: 10.40–12.85) and 3.07% (95% CI: 0.95–9.47), respectively. Our subgroup analysis demonstrated significant and distinct differences in the IS prevalence between the three scores, with a *p*-value < 0.01.

#### Meta-regression for comorbidities and medication use

To account for heterogeneity between studies, meta-regression analysis was used to integrate the prevalence rate of studies that reported the percentage of patients with different comorbidities and medication use. The figures can be found in the Supplementary material. No significant correlation was found for the variables of comorbidities including atrial fibrillation, diabetes mellitus, hypertension, hyperlipidemia, prior myocardial infarction, prior stroke, and medication use of antiplatelets and anticoagulants.

**Figure 6:**
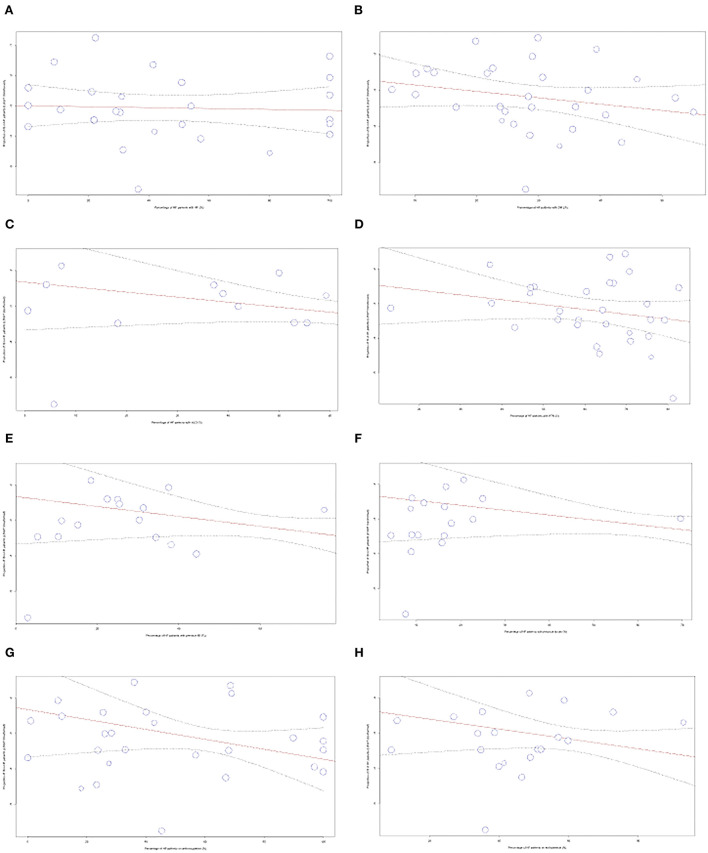
Meta-regression performed for comorbidities and medications used **(A)** AF, **(B)** DM, **(C)** HLD, **(D)** HTN, **(E)** MI, **(F)** Stroke, **(G)** Anticoagulants, **(H)** Antiplatelets.

Meta-regression analysis was used to integrate the prevalence rate of 34 studies that reported the percentage of patients in their study population that were taking anticoagulants, as shown in Figure 6. We observed a significant negative correlation between the variables (*p* = 0.0002). The regression line demonstrated an 82% decrease in IS prevalence from studies with 0–100% anticoagulant usage. We also performed meta-regression to analyze including atrial fibrillation (AF), diabetes mellitus (DM), hyperlipidemia (HLD), hypertension (HTN), previous myocardial infarction (MI), stroke, and antiplatelet usage.

**Figure 7:**
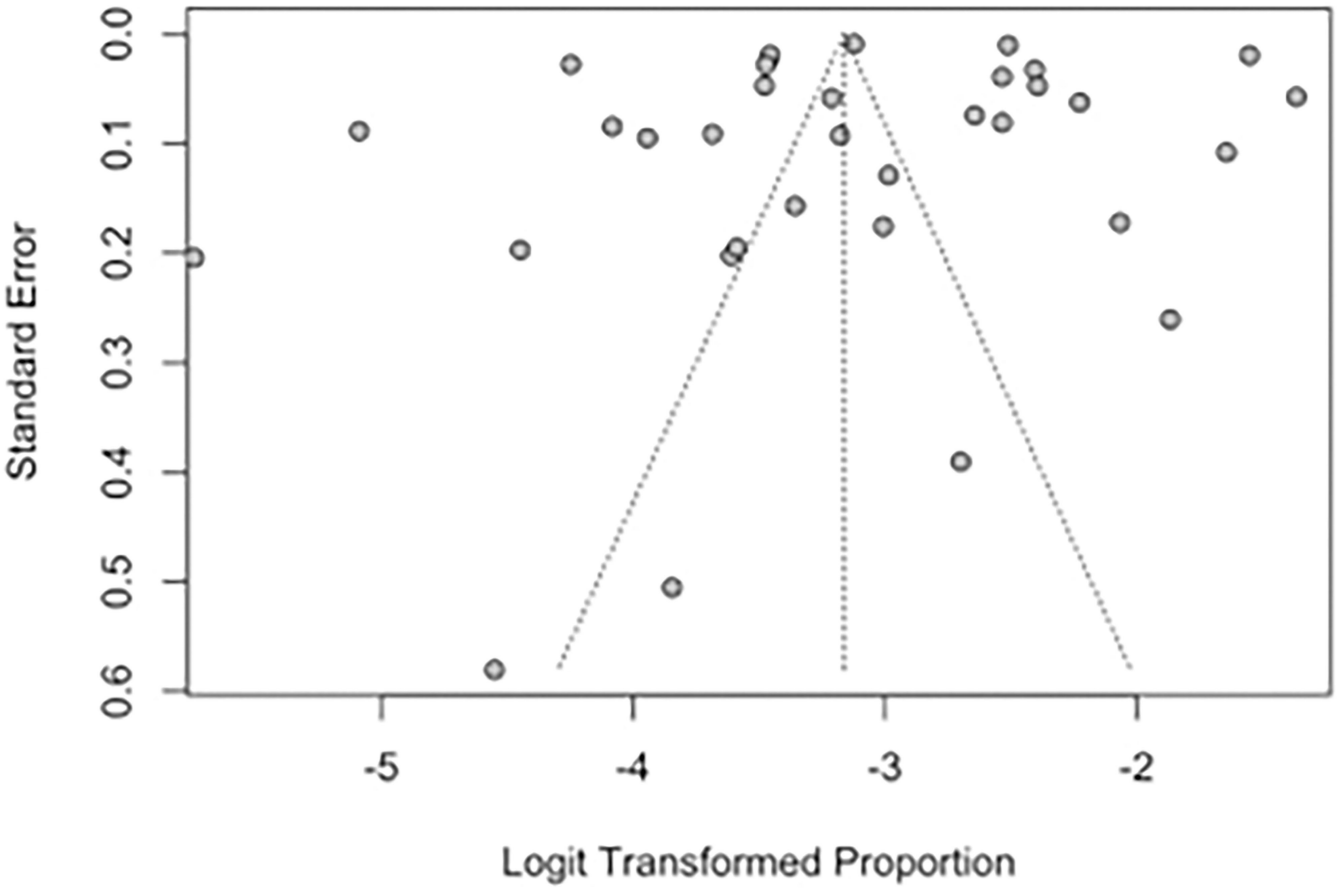
Funnel plot for studies reporting the prevalence of IS in patients with HF.

**Figure 8:**
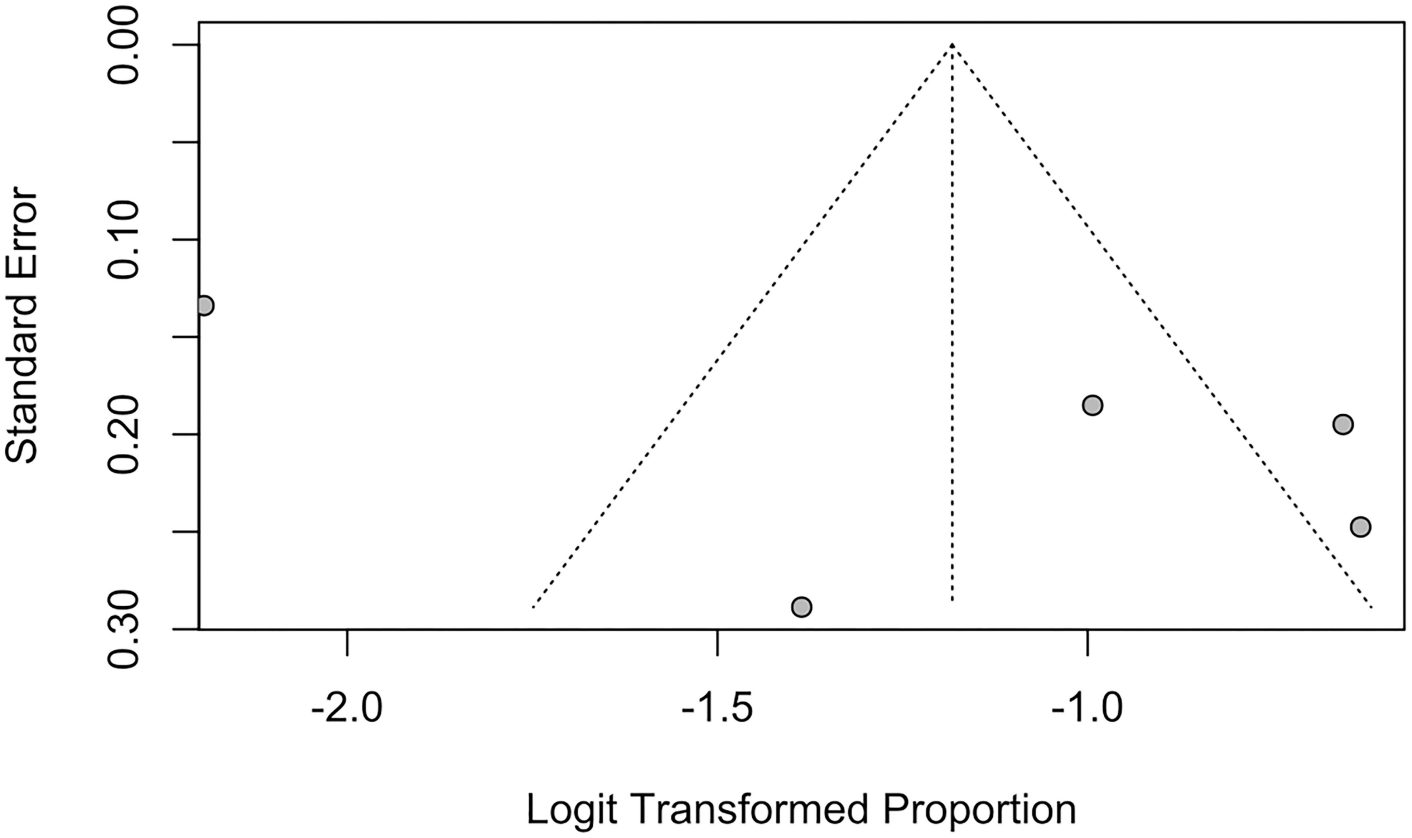
Funnel plot for studies reporting proportion of SBI.

Funnel plots and Egger's regression test were performed to assess for publication bias for studies reporting the prevalence of IS and SBI in patients with HF in Figures 7, 8. There was no evidence of publication bias for the prevalence of IS (p = 0.0568) and prevalence of SBI (p = 0.3543).

## Discussion

Our meta-analysis for the pooled proportion of IS included 35 studies with a combined population of 866,482 patients with HF. The pooled proportion of IS was determined to be 4.06%. There is a statistically significant difference between the pooled proportion of IS in patients with HF with less than and more than 1 year of follow-up (*p* < 0.01). In our subgroup analysis, the pooled proportion of IS in patients with HFpEF and HFrEF was 2.97 and 3.69%, respectively, but no statistically significant difference was observed in both subgroups with different ejection fractions (*p* = 0.48). The prevalence of WMH and SBI in HF is 15.67% and 23.45%, respectively. Subgroup analysis found a statistically significant difference in patients with different Fazekas scores (*p* < 0.01) in which there was a decreasing trend in the prevalence of patients with WMH of a Fazekas score of 1 at 60.57% to Fazekas scores 2 and 3 at 11.57 and 3.07%.

Studies such as Katsanos et al. and Witt et al. have published extensively on HF and the risk of IS recurrence (11, 12). Novelty of our study lies in going beyond IS as the sole endpoint by also analyzing brain lesions leading to the development of IS. WMH and SBI are well-established risk factors of IS (15). Among the various subclinical pathological alterations of the brain parenchyma, WMH is among the most encountered anomalies on MRI (80). Despite mounting evidence of WMH as a risk of IS, few studies have established the relationship between WMH and HF. Despite different pathologies and clinical manifestations, the heart and brain display similarities in vascular anatomy and share common pathophysiological features related to vascular function (81). Small-vessel disease (SVD) is a common pathological mechanism in both WMH and HF. SVD causes coronary microvascular dysfunction, which can lead to both HF and WMH (16). Furthermore, studies have shown that large-vessel atherosclerotic disease and microembolism from unstable plaques also lead to WMH. Our results quantify a pooled proportion of 14.76% patients with HF developing WMH among the included studies. The association between WMH and HF suggests that therapeutic methods to prevent progression to the development of IS should be explored.

In our subgroup analysis of WMH, the Fazekas visual rating scale demonstrated a significant difference in the pooled proportion (*p* < 0.0001). This suggests a distinct heterogeneity among the prevalence of different Fazekas scores among patients with HF. A decreasing trend was observed with the proportion of patients with HF who developed WMH of Fazekas score 1 as 60.57%, Fazekas Score 2 as 11.57%, and Fazekas Score 3 as 3.07%. The scale by Fazekas et al. is an established rating tool to the quantify the severity of white matter involvement (22). Our findings suggest that a large proportion of patients with HF already have white matter involvement, with approximately more than one in every two patients with HF having WMH of a Fazekas score of 1. Furthermore, WMHs have not only been recognized as precursor lesions to stroke but a growing body of evidence strongly suggest these lesions are associated with poorer cognitive performance in patients with HF (82). A previous cross-sectional study by Sauvé et al. (83) suggested the prevalence of cognitive impairment in patients with HF was four times more frequent than in the controls. This combined with our results and the fact that impaired cognition interferes with disease control as HF treatment and monitoring demand a high degree comprehension and self-control (60) shows that WMH in patients with HF is severely under-detected. Hence, there is growing clinical importance for the early screening and recognition of these neuropathological changes.

Furthermore, in our meta-analysis of four studies involving 882 patients, we demonstrated that there is a significantly high prevalence of SBI in patients with HF in approximately one of every four patients. In light of this significant relationship between SBI and HF, there is potential relevance in the detection of SBI among patients with HF to intervene before the development of a clinically overt stroke (84). Currently, there is paucity of information in the literature on the prevention of symptomatic stroke in patients with SBI. To date, the AHA/ASA guideline is the most relevant in recommending primary stroke prevention and vascular risk reduction to prevent symptomatic stroke in individuals with SBI (85). Nevertheless, the high risk of stroke still remains in patients with SBI even after known risk factors are controlled for, and stroke prevention strategies in SBI may be a useful focus in future clinical trials. The high number of HF patients with SBI shown in our study reinforces the notion that patients with SBI should be considered as a high-risk group, and its importance should neither be underestimated nor be under-diagnosed or under-treated.

In our subgroup analysis of HF with varying levels of LVEF, there is a lower prevalence of IS in patients with HFpEF at 2.97% than in patients with HFrEF at 3.69%. This may be consistent with the theory that a lower ejection fraction leads to stasis and the increased formation of LV thrombus (86), which may embolize to the brain. Nevertheless, the relationship between LVEF and IS is not well documented (87). Currently, existing studies including this meta-analysis did not find a significant difference in the prevalence of IS between HFpEF and HFrEF phenotypes at *p* = 0.48 (*p* > 0.05). A meta-analysis by Kotecha et al. found that the incidence of IS between patients with HFpEF and HFrEF was similar (88). By contrast, Greenberg et al. found a significant 1.6-fold higher risk of IS in patients with HFrEF than in patients with HFpEF (31). A possible lack of significant difference may be attributed to the high heterogeneity in studies among patients with HFrEF and HFpEF (I^2^ = 98%).

We postulate that the high heterogeneity found between studies is related to the multiplicity of factors that predispose patients toward IS. Furthermore, limitations of existing studies such as Witt et al. include the inability to draw conclusions regarding patient-level characteristics such as their comorbidities and medication use, citing these as important factors that should be addressed in future research (12). Hence, meta-regression was used in our subgroup analysis to quantify the correlation between factors and account for the heterogeneity in the studies. Our study is, to the best of our knowledge, the first meta-analysis that adopted the use of meta-regression of proportions to not only establish an association but also quantify the correlation between factors that may affect the relationship between HF and IS. Our regression models found no significant correlation was found in comorbidities including atrial fibrillation (AF), diabetes mellitus (DM), hyperlipidemia (HLD), hypertension (HTN), previous myocardial infarction (MI), stroke, and antiplatelet usage.

On the other hand, meta-regression also found a significant 82% decrease in the prevalence of IS from studies with 0% to 100% of anticoagulant treatment in the study population. Our results and the pharmacological mechanism of anticoagulants theorize it as a potentially logical prophylaxis for HF as it prevents venous thrombosis in low-velocity blood flow (5). Nevertheless, there exists a controversy in the current medical scenario with regard to the use of anticoagulation in HF as existing literature is conflicted as to whether the benefits of anticoagulant usage outweigh its risks of life-threatening complications such as hemorrhagic stroke (89). Amid this dilemma, well-considered guidelines provided by the ACC/AHA and ESC still do not strongly recommend the usage of anticoagulants in the treatment of HF (90), and it is only indicated in patients with concomitant AF (91). However, this analysis may be confounded by the fact that studies also included patients with AF who are taking anticoagulants. As such, the incidence of IS in this population will be reduced. Our meta-analysis points to the growing need for future research to adopt multi-variate meta-regression for the proportion of patients with AF and anticoagulant use.

Furthermore, another possible explanation for our heterogeneity would be that a sizeable proportion of our included studies did not specify the type of HFrEF and HFpEF subgroups and were not included in our subgroup analysis, resulting in limited data. It may be helpful for future studies to focus on the specific role of LVEF on the development of IS. Future studies may also attempt to stratify results based on prevalence and severity of carotid plaques and previous vascular surgery, as well as specific cardiovascular risk factors such as valvular heart disease (92), to more accurately target the mechanisms underlying the development of these brain lesions.

Future meta-analysis may also seek to analyze the prevalence and incidence of the different subtypes of ischemic stroke and its relationship with the underlying etiology of HF. While cerebrovascular thromboembolism has been reported as the most common etiology of ischemic stroke, studies have reported varying degrees of prevalence of different subtypes of IS dependent on the etiology of HF. Vemmos et al. had previously reported that non-ischemic etiologies of HF such as valvular heart disease and dilated cardiomyopathy were related to cardioembolism, while HF arising from coronary artery disease was associated with large-vessel atherosclerosis and lacunar strokes.

## Strengths and limitations

Our meta-analysis comprehensively reviewed a significantly large study population of 870,002 patients from 41 studies. While the broad spectrum of included studies is a strength of our meta-analysis, this likely contributed to heterogeneity of study demographics. For instance, most included studies examined Caucasian populations, which limits generalizability to other ethnic groups. Many studies did not publish data on anticoagulant dosages, drug type, or duration administered. The definition of HF and range of LVEF in HFrEF and HFpEF were not standardized across the included studies. There have been varying diagnostic criteria proposed for HFpEF, which could have contributed to heterogeneity in the patients included in studies, trials, and registries (90). Furthermore, some included studies chose to classify patients with LVEF within the range (40% ≤ LVEF ≤ 50%) under HFrEF, even though the most widely used classification of HFrEF would be LVEF <40% (26, 33, 46, 55, 93, 94). Future studies should use the latest available guidelines to standardize the definition of the type of heart failure (95).

## Conclusion

In conclusion, we estimated that four in 100 patients with HF on average suffer from an ischemic stroke, with a higher prevalence reported in studies after more than 1 year of follow-up. However, we were unable to determine any significant difference in the prevalence of IS between patients with HFpEF and HFrEF, which may have been attributed to high between-study heterogeneity and therefore may warrant future studies and reviews in this area. Our most significant finding would be the high prevalence of WMHs and SBIs in patients with HF, with nearly one in every two HF patients with WMH of a Fazekas score 1, and one in every four HF patients with SBI. This highlights the potential under-detection and under-treatment of these conditions, given that these cerebrovascular lesions predispose patients to significant cognitive impairment and risk of future strokes in the long run.

## Data availability statement

The original contributions presented in the study are included in the article/Supplementary material, further inquiries can be directed to the corresponding author/s.

## Author contributions

ST and CH contributed to conception and design of the study. YHT and YNT performed the statistical analysis. ST and CH wrote the first draft of the manuscript. All authors contributed to manuscript revision, read, and approved the submitted version.

## Funding

This research was sponsored by the National Medical Research Council (NMRC), Singapore (NMRC/MOH-TA19Nov-0003). BT is supported by the Ministry of Health, Singapore (MOH) – Healthcare Research Scholarship – Master of Clinical Investigation (MCI). LY is supported by National Medical Research Council – NMRC/MOH-TA19Nov-003.

## Conflict of interest

The authors declare that the research was conducted in the absence of any commercial or financial relationships that could be construed as a potential conflict of interest.

## Publisher's note

All claims expressed in this article are solely those of the authors and do not necessarily represent those of their affiliated organizations, or those of the publisher, the editors and the reviewers. Any product that may be evaluated in this article, or claim that may be made by its manufacturer, is not guaranteed or endorsed by the publisher.
