# Supplementary material for: Prevalence and incidence of stroke, white matter hyperintensities, and silent brain infarcts in patients with chronic heart failure: A systematic review, meta-analysis, and meta-regression
Source: Front Cardiovasc Med. 2022 Sep 15;9:967197. doi: 10.3389/fcvm.2022.967197 (PMC9520068; doi:10.3389/fcvm.2022.967197)
Supplement: Supplementary file 1 [file Data_Sheet_1.docx]

# Appendix A: Search Method

(“stroke” OR “cerebrovascular disease” OR “cerebral artery disease” OR “cerebral ischaemia” OR “cerebrovascular accident” OR “brain infarction” OR “brain ischaemia” OR “ischemic stroke” OR “cerebrovascular attack” OR “white matter hyperintensities” OR “white matter hyperintensity” OR “white matter lesions” OR “white matter lesion” OR “leukoaraiosis” OR “silent brain infarcts” OR “silent cerebral infarction” OR “silent ischaemic lesions” OR “silent lacunes”) AND (“heart failure”) AND (“incidence” OR “prevalence”) AND (“trial” OR “cohort” OR “case control” OR “case-control” OR “observational” OR “longitudinal” OR “study” OR “cross sectional” OR “cross-sectional”)

# Appendix B: Supplementary Tables and Figures

**Table S1. Newcastle-Ottawa Scale for Cohort Studies**

| **Study** | | | **Selection** | | | **Comparability** | | | **Outcome/Exposure** | | |
| --- | --- | --- | --- | --- | --- | --- | --- | --- | --- | --- | --- |
| **Author, Year** | **Representativeness of the exposed cohort** | **Selection of non-exposed cohort** | | **Ascertainment of exposure** | **Outcome of interest not present at start** | | **Comparability of cohorts** | **Assessment of outcome** | | **Follow up long enough for outcomes** | **Adequacy of follow up of cohorts** |
| **PROSPECTIVE STUDIES** | | | | | | | | | | | |
| Adelborg et al. 2017 | ★ | ★ | | ★ | ★ | | ★★ | ★ | | ★ | NR |
| Alberts et al. | ★ | ★ | | ★ | ★ | | ★★ | ★ | | ★ | NR |
| Berkovitch et al. 2019 | 1 hopsital only | ★ | | ★ | ★ | | ★★ | ★ | | ★ | NR |
| Frey et al. 2018 | Only patients from Cognition Matters HF | Did not describe non-exposed cohort | | ★ | ★ | | ★★ | ★ | | ★ | NR |
| Hamatani et al. 2018 | ★ | NR | | ★ | ★ | | ★ | ★ | | ★ | NR |
| Hjalmarsson et al. 2021 | ★ | ★ | | ★ | ★ | | ★★ | ★ | | ★ | NR |
| Iguchi et al. 2018 | ★ | NR | | ★ | ★ | | ★★ | ★ | | ★ | 10% loss to follow up |
| Kang et al. 2017 | ★ | ★ | | ★ | ★ | | ★★ | ★ | | ★ | NR |
| Kim et al. 2017 | ★ | NR | | ★ | ★ | | ★★ | ★ | | ★ | NR |
| Komori et al. 2008 | Only patients at the Jichi Medical University | NR | | No criteria for diagnosis of CHF | ★ | | ★★ | ★ | | ★ | ★ (6 patients lost to follow up) |
| Kozdag et al. 2008 | Did not state the source of their population | Volunteers | | ★ | ★ | | ★★ | ★ | | ★ | NR |
| Lip et al. 2012 | ★ | NR | | ★ | ★ | | ★★ | ★ | | ★ | NR |
| Melgaard et al. 2015 | ★ | ★ | | ★ | ★ | | ★ | ★ | | ★ | NR |
| Nakano et al. 2021 | ★ | ★ | | ★ | ★ | | ★★ | ★ | | ★ | NR |
| Oliveira et al. 2018 | 1 referral hospital only | ★ | | ★ | ★ | | ★★ | ★ | | ★ | NR |
| Qualls et al. 2013 | ★ | NR | | ★ | ★ | | ★★ | ★ | | ★ | NR |
| Shintani et al. 2019 | ★ | NR | | ★ | ★ | | ★★ | ★ | | ★ | 13% loss to follow up |
| Siachos et al. 2005 | Only patients evaluated at a medical university | NR | | ★ | ★ | | ★★ | ★ | | ★ | NR |
| Stegmann et al. 2020 | ★ | ★ | | ★ | ★ | | ★★ | ★ | | ★ | NR |
| Tai et al. 2020 | ★ | ★ | | ★ | ★ | | ★★ | ★ | | ★ | NR |
| Tütüncü et al. 2020 | ★ | ★ | | ★ | ★ | | ★ | ★ | | ★ | NR |
| Vemmos et al.2011 | Only patients at the Alexandra University hospital in Athens | ★ | | ★ | ★ | | ★★ | ★ | | ★ | ★ 240 patients (8.4%) were lost to follow-up |
| Witt et al. 2006 | ★ | NR | | ★ | ★ | | ★★ | ★ | | ★ | NR |
| Zhirov et al. 2019 | ★ | NR | | ★ | ★ | | ★★ | ★ | | ★ | NR |
| Zhou et al. 2021 | Only patients at the First Affiliated Hospital of Wenzhou Medical University | NR | | ★ | ★ | | ★★ | ★ | | ★ | 198 patients have missing follow-up data |
| **RETROSPECTIVE STUDIES** | | | | | | | | | | | |
| Berger et al. 2018 | ★ | ★ | | ★ | ★ | | ★★ | ★ | | ★ | NR |
| Chou et al. 2020 | ★ | ★ | | ★ | ★ | | ★★ | ★ | | ★ | ★ |
| Davis et al. 1996 | ★ | ★ | | ★ | ★ | | ★ | ★ | | ★ | NR |
| de Peuter et al. 2010 | ★ | NR | | ★ | ★ | | ★★ | ★ | | ★ | ★ |
| Friberg etl al. 2018 | ★ | NR | | ★ | ★ | | ★★ | ★ | | ★ | NR |
| Greenberg et al. 2018 | ★ | ★ | | ★ | ★ | | ★★ | ★ | | ★ | NR |
| Kondo et al. 2016 | Did not state the source of their population | Did not state source of population | | ★ | ★ | | ★★ | ★ | | ★ | NR |
| Loh et al. 1997 | ★ | ★ | | ★ | ★ | | ★★ | ★ | | ★ | NR |
| Merkler et al. 2019 | ★ | ★ | | ★ | ★ | | ★★ | ★ | | ★ | NR |
| Nakayama et al. 2020 | Only patients at the from Sagamihara National Hospital | NR | | ★ | ★ | | ★★ | ★ | | ★ | NR |
| Wolsk et al. 2015 | ★ | NR | | ★ | ★ | | ★ | ★ | | ★ | NR |
| **RANDOMIZED CLINICAL TRIAL** | | | | | | | | | | | |
| Chi et al. 2017 | ★ | ★ | | ★ | **★** | | ★★ | ★ | | ★ | NR |
| McMurray et al. 2013 | ★ | ★ | | ★ | ★ | | ★★ | ★ | | ★ | NR |
| Mehra et al. 2019 | ★ | ★ | | ★ | ★ | | ★★ | ★ | | ★ | NR |
| Tseng et al. 2017 | Only patients from Mayo Clinic | NR | | ★ | ★ | | ★ | ★ | | ★ | NR |
| Yusuf et al. 2003 | ★ | ★ | | ★ | ★ | | ★★ | ★ | | ★ | ★ (3 patients loss to follow-up) |

**Table S2. Baseline Characteristics of Included Studies**

| **Study Name** | **Year** | **HF Definition** | **Total Number of HF Patients** | **Gender Distribution (M)** | **Age/ year** | **Follow-up Period/ year** | **Concurrent AF** | **DM** | **HTN** | **HLD** | **Previous MI** | **Previous Stroke** | **Anti-Platelet** | **Anti-Coagulation** | **Statins** |
| --- | --- | --- | --- | --- | --- | --- | --- | --- | --- | --- | --- | --- | --- | --- | --- |
| Adelborg et al. | 2017 | ICD | 289,353 | 51.96% | 77.0 | 0 to 30 days | 10.70% | 10.10% | 13.20% | 0.60% | 15.20% | NR | NR | NR | NR |
| Alberts et al. | 2010 | European Society of Cardiology (ESC) guidelines | 1,247 | NR | NR | 5.00 | NR | NR | NR | NR | NR | NR | NR | NR | NR |
| Berger et al. | 2019 | Clinical Diagnosis | 66,414 | 50.00% | 68.0 | 3.00 | 21.70% | 36.00% | 75.80% | 55.50% | 5.30% | 17.90% | NR | NR | NR |
| Berkovitch et al. | 2019 | European Society of Cardiology (ESC) guidelines | 2,922 | 50.00% | 79.0 | 1.00 | 100.00% | 30.66% | 60.37% | 38.98% | NR | NR | NR | 89.84% | NR |
| Chi et al. | 2017 | Clinical Diagnosis | 7,513 | 44.86% | 76.0 | 77 days | 36.43% | 27.91% | 81.23% | 5.70% | NR | 10.40% | 57.03% | 100% | NR |
| Chou et al. | 2020 | ICD | 12,179 | 45.40% | 66.6 | 5.95 | 0% | 12% | 66.90% | 37.20% | NR | NR | NR | 1.03% | NR |
| Davis et al. | 1996 | Clinical Diagnosis | 618 | 61.30% | 66.0 | NR | NR | NR | NR | NR | NR | NR | NR | NR | NR |
| de Peuter et al. | 2011 | ICD | 20,870 | 49% | 75.0 | 2.00 | NR | NR | NR | NR | NR | NR | NR | NR | NR |
| Frey et al. | 2018 | European Society of Cardiology (ESC) guidelines | 148 | 84.50% | 65.0 | 5.00 | 21.60% | 29.73% | 79.72% | 72.00% | 25.68% | NR | 57.40% | 30.40% | NR |
| Friberg etl al. | 2018 | Clinical Diagnosis | 92,532 | 54.20% | 79.2 | 1.00 | 100.00% | 23.80% | 53.50% | NR | 31.30% | 16.40% | 52.00% | 45.30% | NR |
| Greenberg et al. | 2019 | Clinical Diagnosis | 7,005 | 52.00% | 73.5 | 1.00 | 22.30% | NR | NR | NR | 2.90% | 7.60% | 10.60% | 25.50% | NR |
| Hamatani et al. | 2018 | Framingham Heart Study Criteria | 721 | 60.00% | 76.0 | 30 days | 54.00% | 38.00% | 75.00% | 53.00% | 25.00% | 25.00% | 36.00% | 33.00% | NR |
| Hjalmarsson et al. | 2021 | Clinical Diagnosis | 15,425 | 61.30% | 73.0 | 1.67 | NR | 28.90% | 58.50% | NR | NR | 9.00% | 72.80% | 26.10% | NR |
| Iguchi et al. | 2018 | NYHA Class II, III or IV | 338 | 51.50% | 78.4 | 3.00 | 100.00% | 29.00% | 70.70% | 42.00% | 10.40% | 22.80% | NR | 68.00% | NR |
| Kang et al. | 2017 | ICD | 5,746 | 41.00% | 70.0 | 5.60 | NR | 22.60% | 66.00% | NR | 11.20% | 16.30% | 51.10% | 10.20% | NR |
| Kim et al. | 2017 | ICD | 1,869 | NR | 43.9 | 5.00 | 100.00% | NR | NR | NR | NR | NR | NR | NR | NR |
| Komori et al. | 2008 | Clinical Diagnosis | 111 | 78.20% | 67.5 | 1.50 | 31% | 45.94% | NR | NR | NR | NR | NR | 40% | NR |
| Kondo et al. | 2016 | Framingham Heart Study Criteria | 127 | 76.40% | 64.0 | 8.40 | NR | 39.37% | 46.74% | NR | NR | NR | 33.85% | 42.80% | 51.43% |
| Kozdag et al. | 2008 | NYHA Class II, III or IV | 72 | 73.61% | 62.0 | 5.00 | NR | 30.56% | 55.56% | 25.00% | NR | NR | 77.78% | NR | NR |
| Lip et al. | 2012 | ICD | 1,309 | 37.80% | 67.1 | 1.00 | 0.00% | 6.30% | 37.10% | NR | 34.40% | NR | 8.94% | 69.00% | 5.81% |
| Loh et al. | 1997 | European Society of Cardiology (ESC) guidelines | 2,231 | 82.52% | 59.0 | 1.75 | NR | 10.17% | 37.52% | NR | 37.52% | NR | 58.72% | 28.33% | 58.72% |
| McMurray et al. | 2013 | Clinical Diagnosis | 5,943 | 67.50% | 68.0 | 1.50 | 100.00% | 25.98% | 82.61% | NR | 22.46% | 16.69% | 35.15% | 100.00% | 40.16% |
| Mehra et al. | 2019 | Clinical Diagnosis | 5,022 | 57.37% | 66.4 | 1.70 | 0% | 40.86% | 75.32% | NR | 75.73% | 9.02% | 93% | 97% | NR |
| Melgaard et al. | 2015 | ICD | 42,987 | 55.30% | 74.0 | 1.00 | 21.90% | 16.68% | 43.09% | NR | 18.41% | 8.75% | 48.64% | 0.00% | 28.62% |
| Merkler et al. | 2019 | ICD | 7,848 | 65.58% | 54.0 | 2.70 | 30.45% | 52.14% | 79.22% | NR | NR | NR | NR | NR | NR |
| Nakano et al. | 2021 | ICD | 92,573 | 48.20% | 81.3 | NR | NR | 28.6%% | 53.90% | 18.30% | NR | NR | 38.70% | 23.71% | NR |
| Nakayama et al. | 2020 | American College of Cardiology | 191 | 46.07% | 75.0 | 1.00 | 41.88% | 24.08% | 62.83% | NR | NR | NR | NR | NR | NR |
| Oliveira et al. | 2018 | European Society of Cardiology (ESC) guidelines | 75 | 56.00% | 61.8 | 1.50 | 17.30% | 26.70% | 80.00% | NR | NR | NR | 54.70% | 25.30% | NR |
| Qualls et al. | 2013 | ICD | 8,558 | 50.84% | 78.1 | 1.00 | 57.20% | 35.52% | 70.76% | NR | 30.32% | 20.69% | Aspirin: 26.91%, Clopidogrel 3.97% | 100% | 39.10% |
| Shintani et al. | 2020 | Framingham Heart Study Criteria | 950 | 61.00% | 77.0 | 2.00 | 51.00% | 55.07% | 71.00% | 50.00% | NR | NR | 45.00% | 67.00% | NR |
| Siachos et al. | 2005 | Clinical Diagnosis | 117 | 74.00% | 51.0 | 1.00 | NR | 21.00% | 31.00% | NR | NR | NR | 26.00% | 27.00% | NR |
| Stegmann et al. | 2021 | Clinical Diagnosis | 2,490 | 54.00% | 64.0 | NR | NR | NR | NR | NR | NR | NR | NR | NR | NR |
| Tai et al. | 2020 | Clinical Diagnosis | 18,373 | 50.10% | 75.0 | 14.00 | 8.60% | 29.90% | 58.20% | 4.20% | NR | 69.60% | NR | 27.50% | NR |
| Tseng et al. | 2017 | ICD | 287 | 84% | 68.2 | 0.85 | 80.10% | 33.40% | 69.70% | NR | NR | NR | NR | 100% | NR |
| Tütüncü et al. | 2020 | Clinical Diagnosis | 2,248 | 73.44% | 71.0 | 1.00 | 31.45% | 43.46% | 75.98% | 59.25% | NR | NR | 39.90% | 56.80% | NR |
| Vemmos et al. | 2012 | European Society of Cardiology (ESC) guidelines | 2,904 | 67.10% | 70.4 | 3.86 | 50.90% | NR | 63.50% | NR | NR | NR | 49.00% | 36.00% | 14.20% |
| Witt et al. | 2006 | Framingham Heart Study Criteria | 630 | 46.10% | 76.0 | 4.30 | 41.42% | 19.84% | 66.03% | NR | 25.39% | 11.74% | 34.76% | 18.10% | NR |
| Wolsk et al. | 2015 | European Society of Cardiology (ESC) guidelines | 136,545 | 53.00% | 73.3 | 3.60 | NR | 13.10% | 47.80% | NR | NR | NR | NR | NR | 24.90% |
| Yusuf et al. | 2003 | NYHA Class II, III or IV | 3,023 | 59.86% | 67.1 | 3.00 | 29.14% | 28.35% | 64.27% | NR | 44.33% | 8.87% | 59.84% | 23.34% | 39.37% |
| Zhirov et al. | 2019 | Clinical Diagnosis | 1,003 | 56.40% | 68.0 | 1.00 | 100.00% | 24.60% | 65.10% | NR | 38.10% | 15.80% | 46.50% | 68.60% | 60.40% |
| Zhou et al. | 2021 | Clinical Diagnosis | 9,485 | 70.90% | 66.4 | 1.38 | 21.10% | 21.70% | 46.90% | 7.20% | NR | 4.30% | 41.40% | 11.50% | NR |

**Figure S3 Forest Plot of IS in HFpEF patients at less and more than 1 year of follow-up**

**
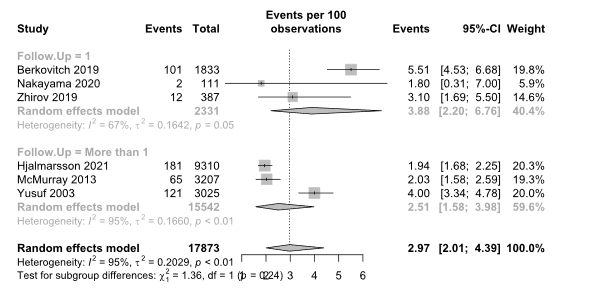
**

**Figure S4 Forest Plot of IS in HFrEF patients at less and more than 1 year of follow-up**

**
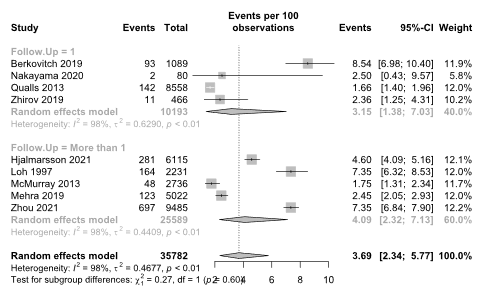
**

**Figure S5. Forest Plot of Hazard Ratios for Ischemic Stroke in HF Patients**

**
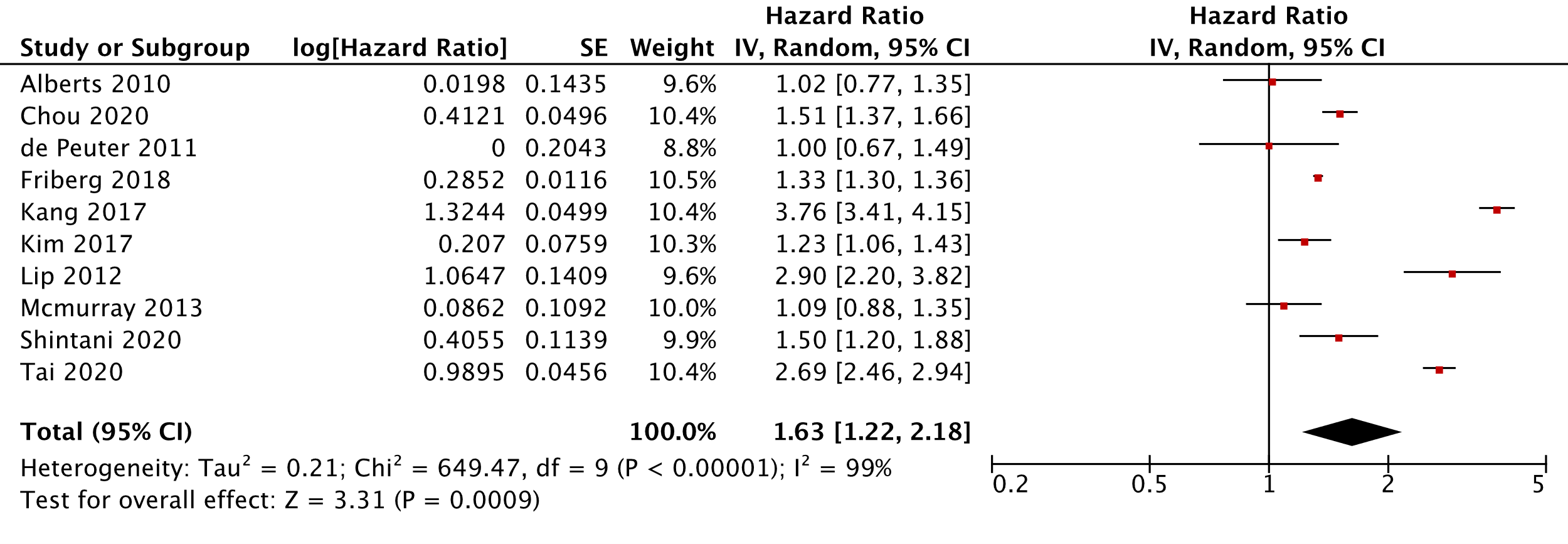
**

**Figure S6. Forest Plot of Risk Ratios for Ischemic Stroke in HF Patients**

**
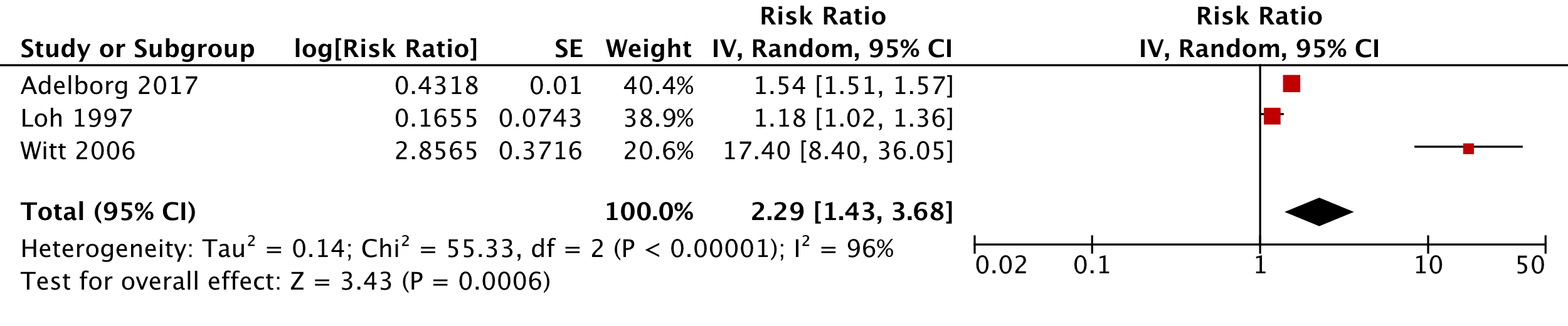
**

**Figure S7. Forest Plot of Prevalence of SBI in HF Patients for more than 1 year of follow-up**

**
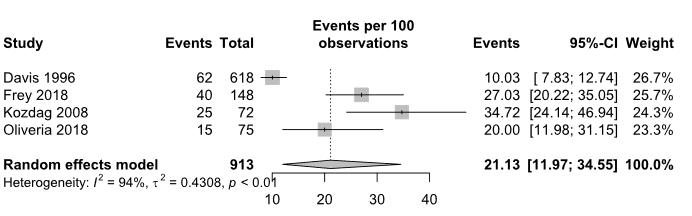
**

**Figure S8. Meta-regression of LOGIT transformed proportion of IS in HF patients against percentage of HF patients with atrial fibrillation (AF)**

**
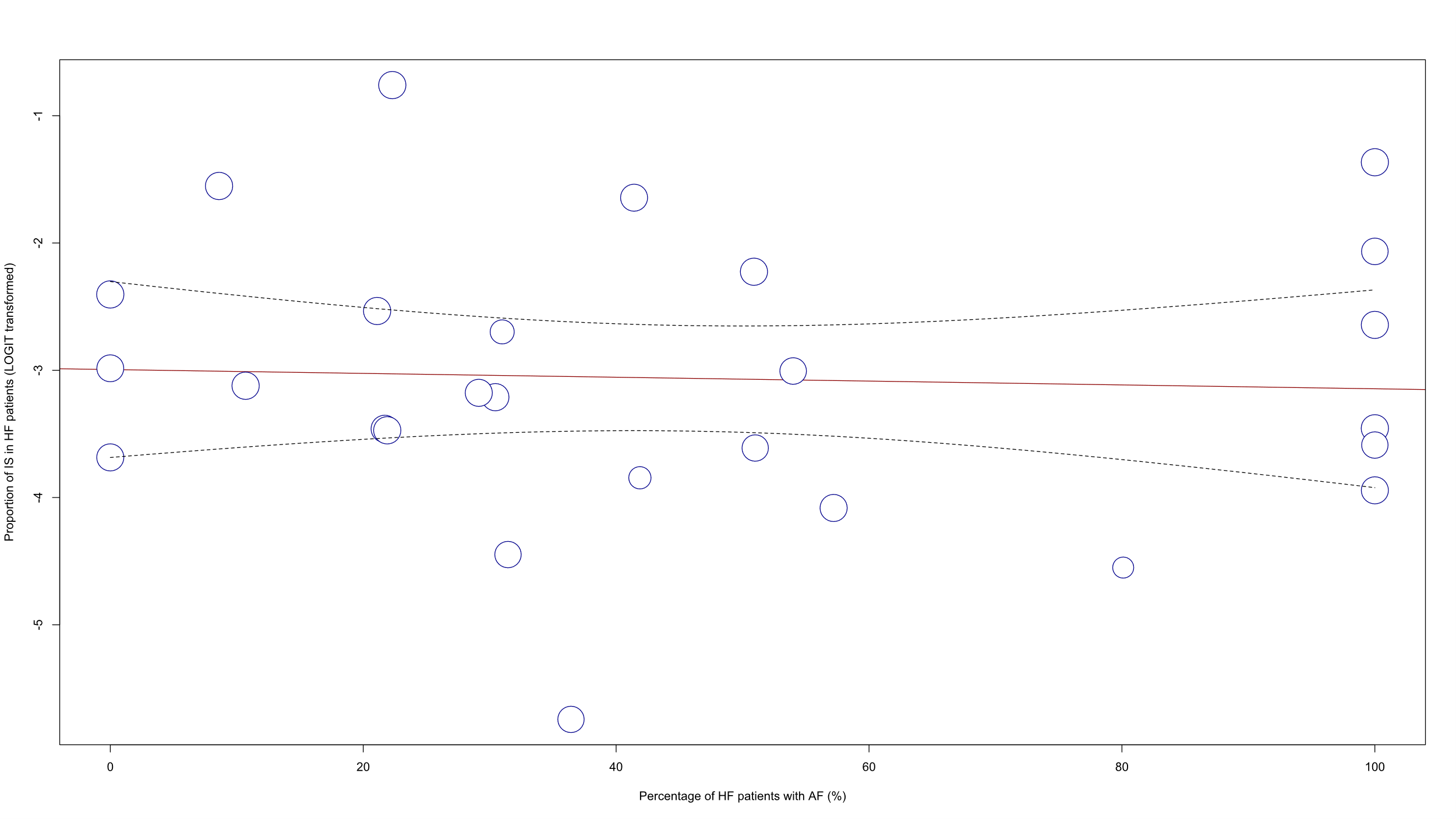
**

**Figure S9. Meta-regression of LOGIT transformed proportion of IS in HF patients against percentage of HF patients on anticoagulation**

**
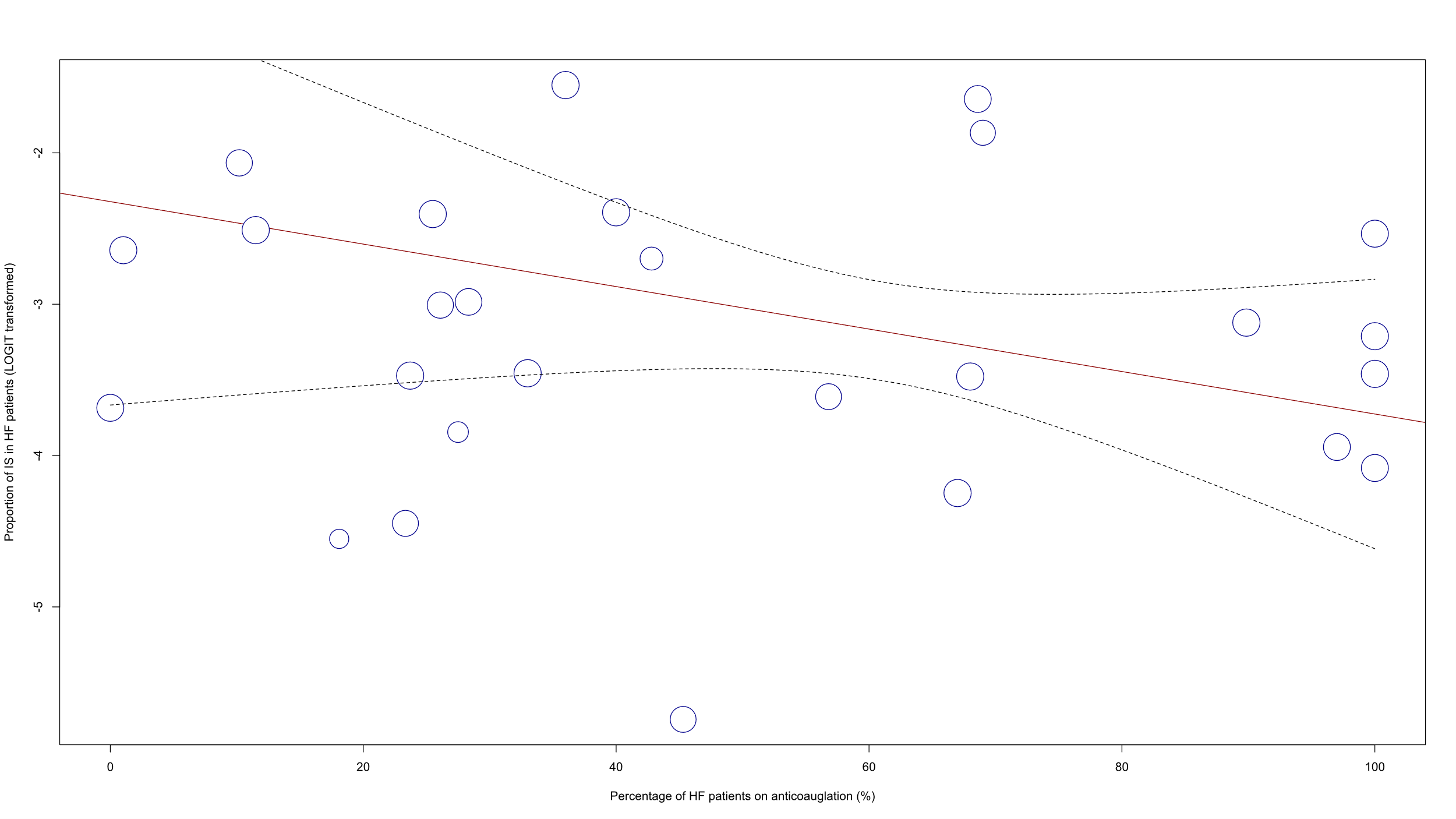
**

**Figure S10. Meta-regression of LOGIT transformed proportion of IS in HF patients against percentage of HF patients on anti-platelets**

**
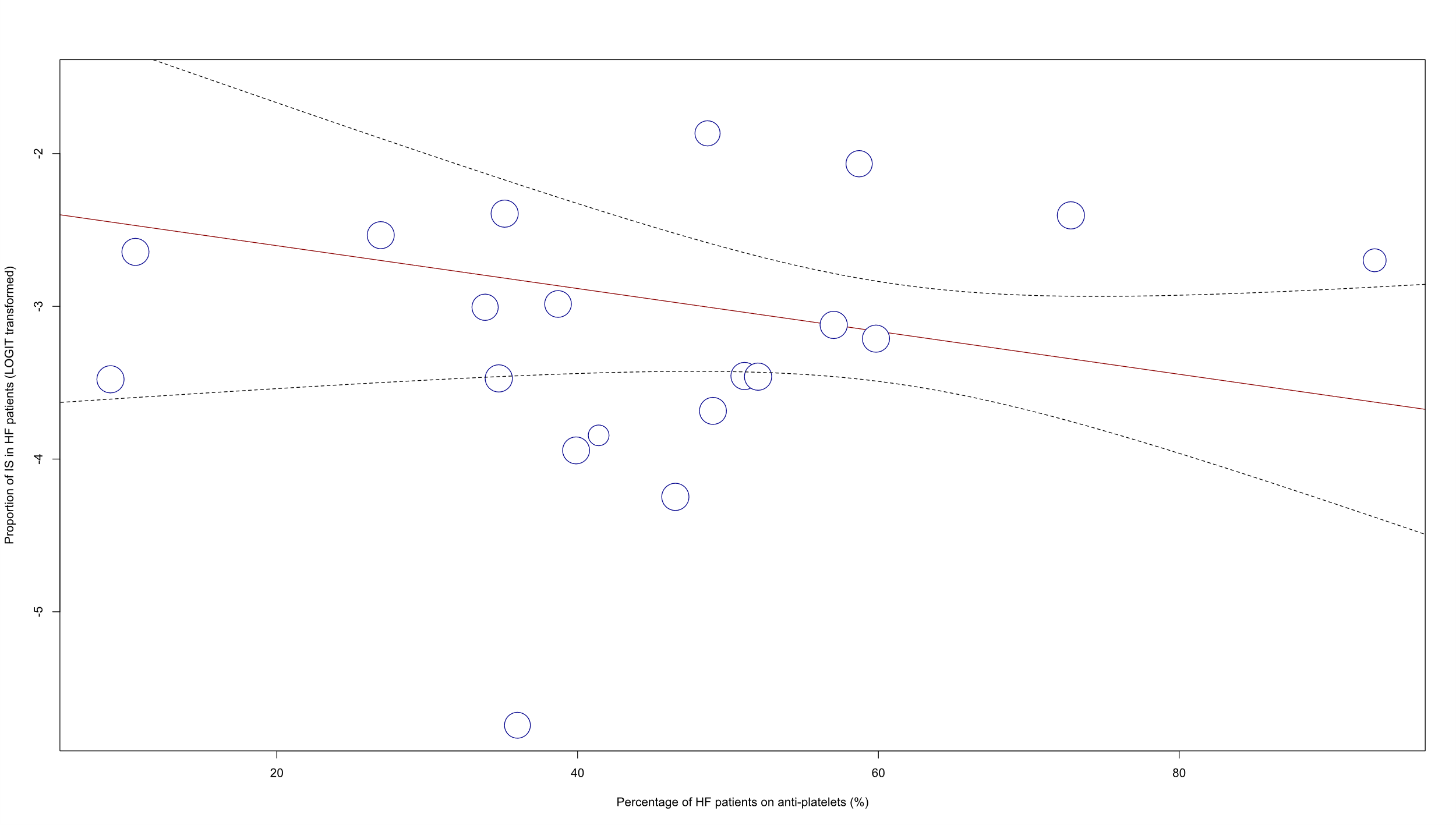
**

**Figure S11. Meta-regression of LOGIT transformed proportion of IS in HF patients against percentage of HF patients with diabetes mellitus (DM)**

**
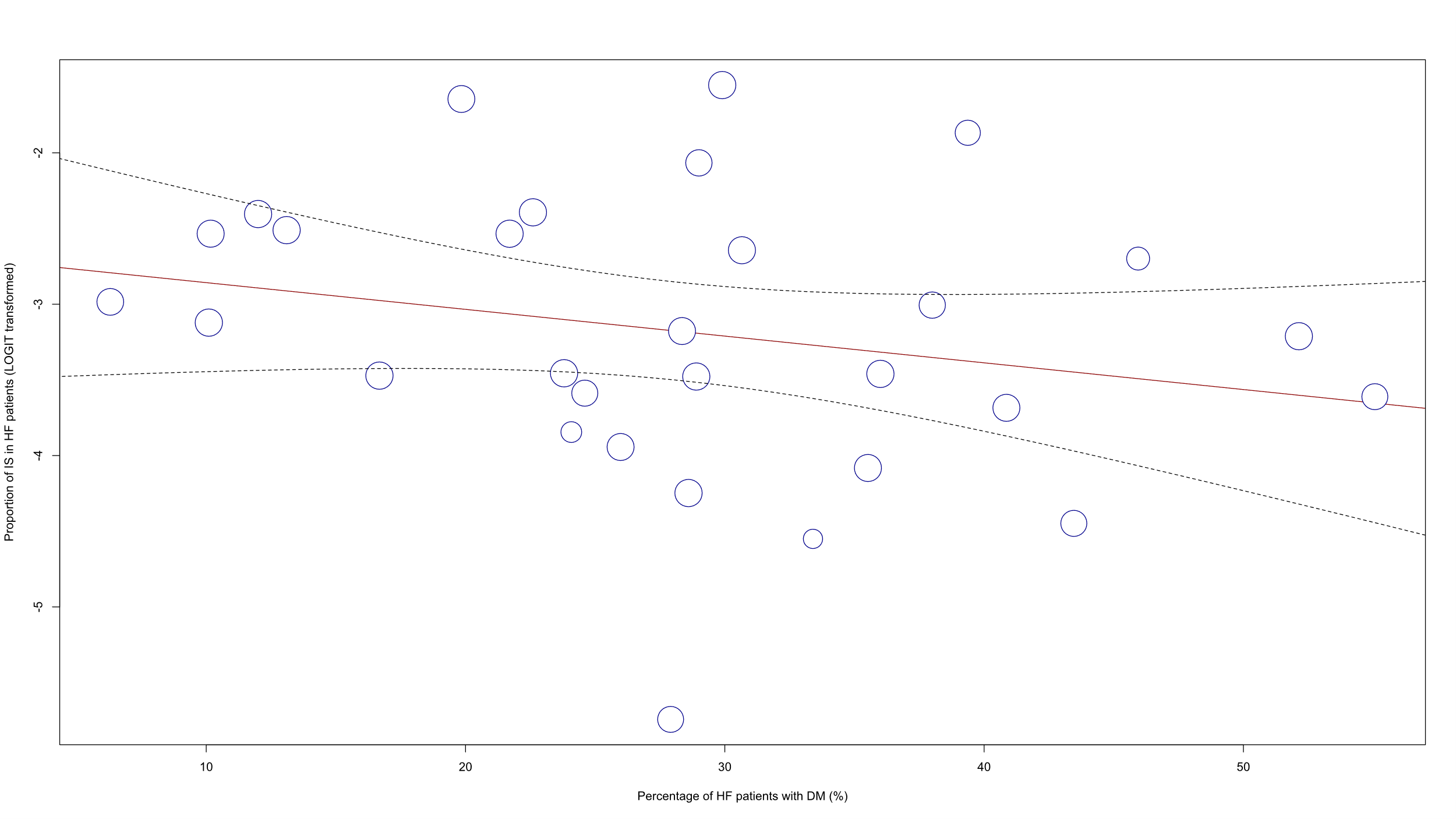
**

**Figure S12. Meta-regression of LOGIT transformed proportion of IS in HF patients against percentage of HF patients with hyperlipidemia (HLD)**

**
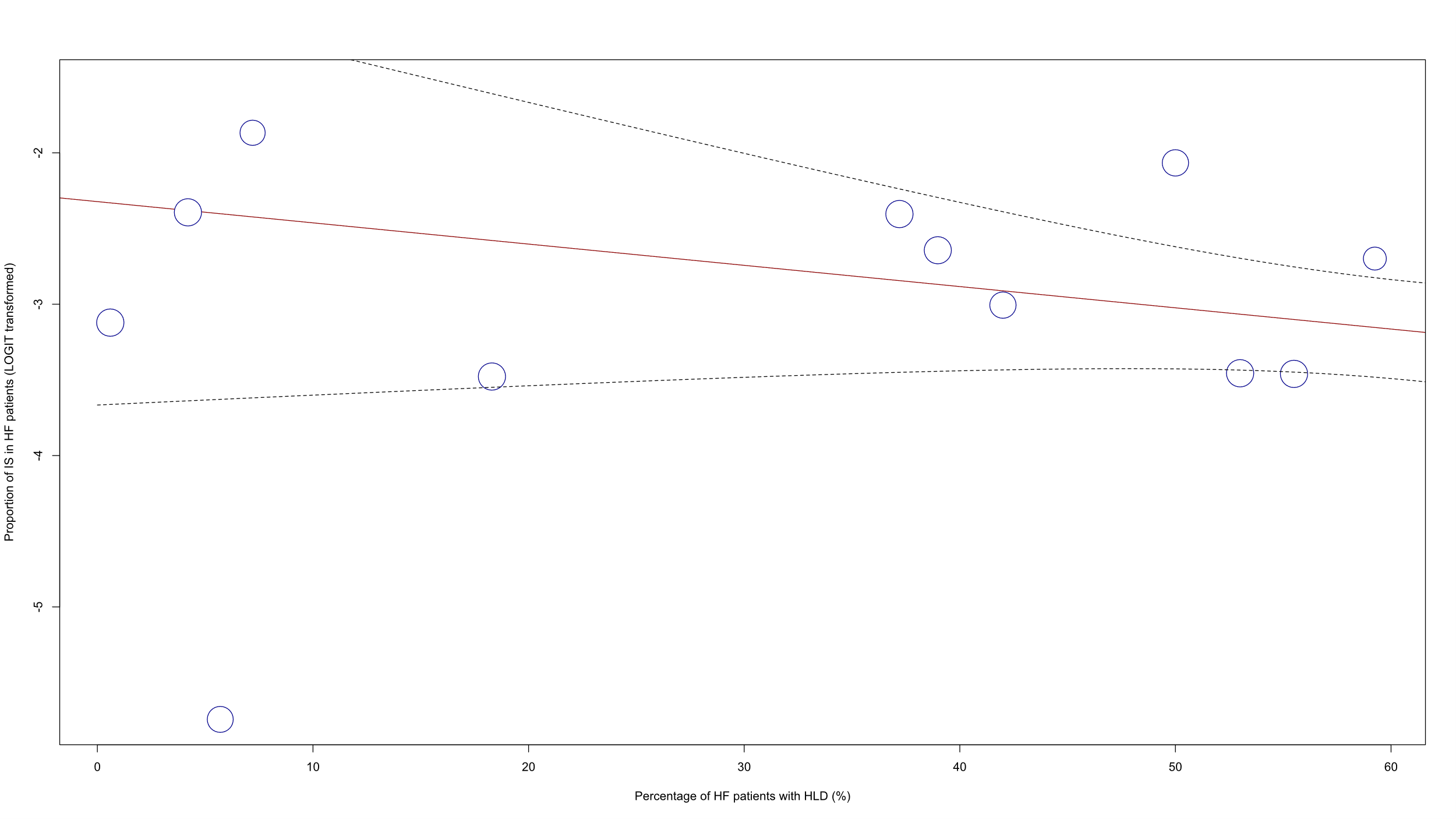
**

**Figure S13. Meta-regression of LOGIT transformed proportion of IS in HF patients against percentage of HF patients with hypertension (HTN)**

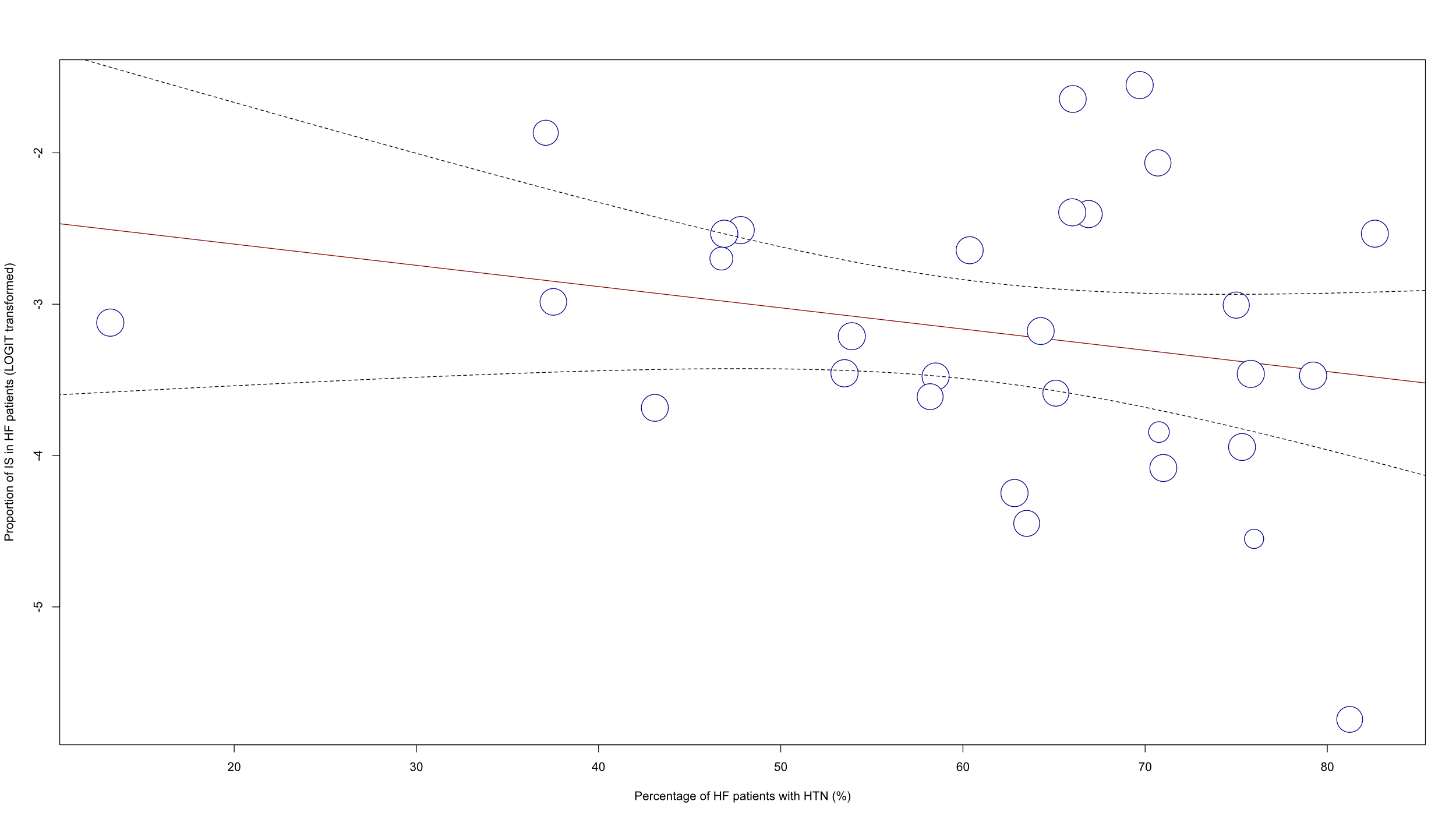


**Figure S14. Meta-regression of LOGIT transformed proportion of IS in HF patients against percentage of HF patients with previous myocardial infarction (MI)**

**
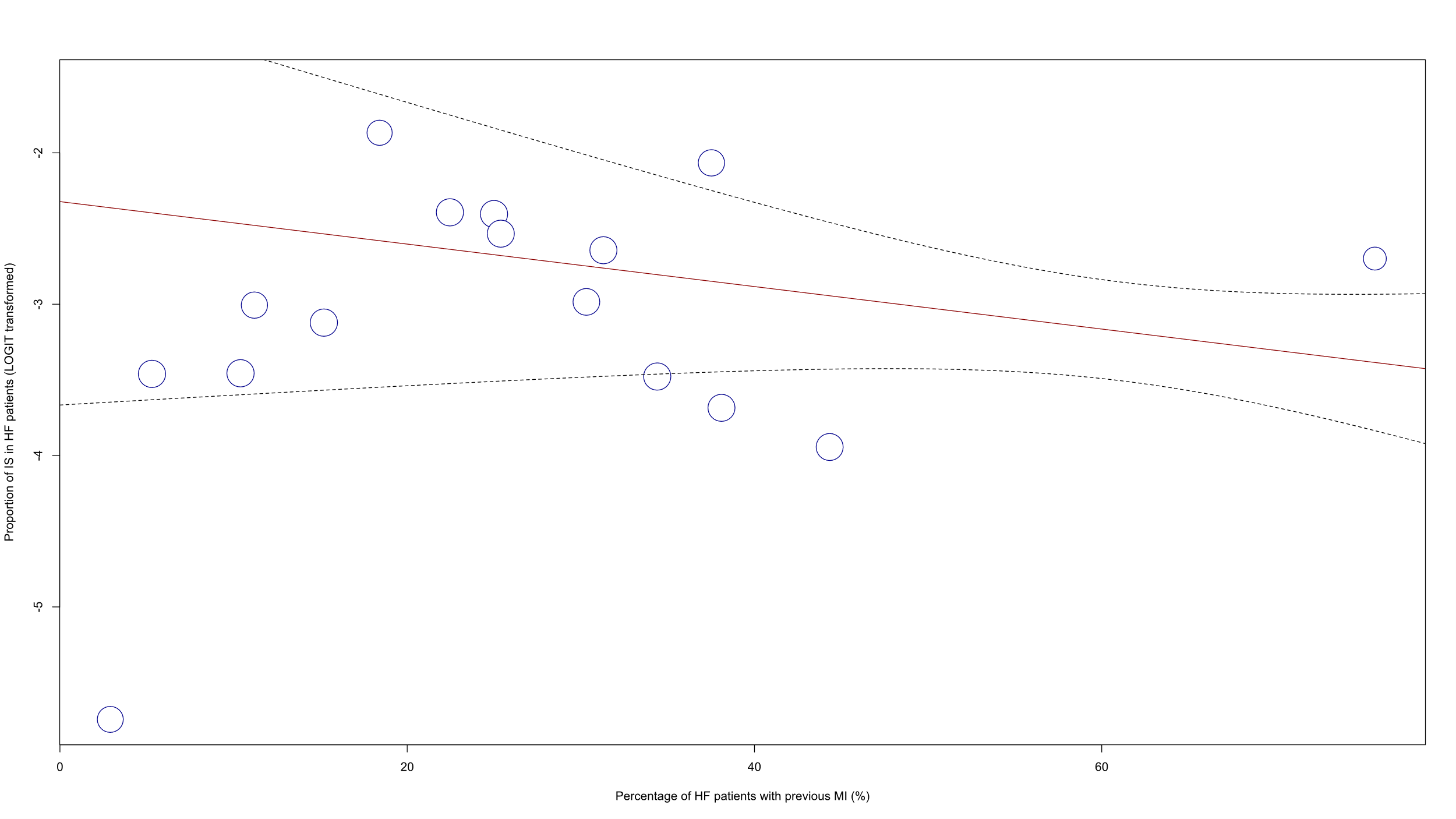
**

**Figure S15. Meta-regression of LOGIT transformed proportion of IS in HF patients against percentage of HF patients with previous stroke**

**
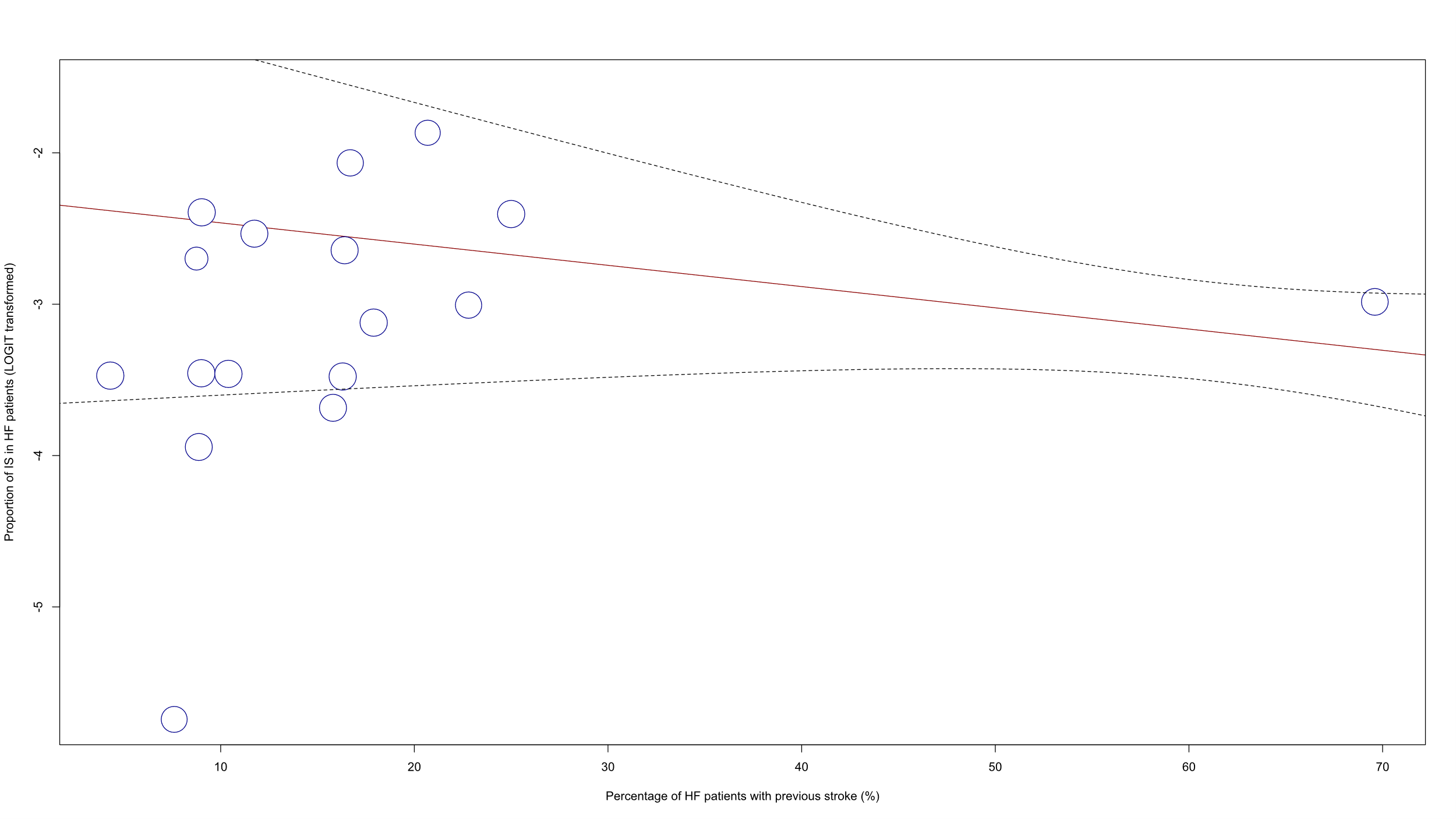
**
